# The Revolution in Surgery That Saves Millions of Lives

**DOI:** 10.3390/jcm15124476

**Published:** 2026-06-09

**Authors:** Camran Nezhat, Barbara Page, Zoë Pennington, Rana Khaloghli, Lillian Niehaus, Zahra Najmi

**Affiliations:** 1 Camran Nezhat Institute Center for Minimally Invasive and Robotic Surgery, Woodside, CA 94061, USA; bjpage.research@gmail.com (B.P.); rananicolek@gmail.com (R.K.); lily@camrannezhatinstitute.com (L.N.); zahra.najmi@camrannezhatinstitute.com (Z.N.); 2 Stanford University, Medical Center, Palo Alto, CA 94305, USA; 3 University of California, San Francisco, CA 94143, USA

**Keywords:** minimally invasive surgery, MIS, video-assisted endoscopic surgery, video-assisted laparoscopy, laparoscopic surgery, robotic-assisted endoscopic surgery, surgical innovation, surgical outcomes, perioperative mortality, postoperative morbidity, enhanced recovery, oncologic equivalence, patient safety, surgical education, technology adoption, operative visualization, health economics, quality of life, evidence-based surgery, surgical training, historical perspective, Camran Nezhat

## Abstract

The introduction of minimally invasive surgery (MIS) marked a turning point in the history of medicine, driving one of the sharpest declines in surgical mortality and morbidity ever recorded—saving millions of lives and sparing an estimated one billion patients the suffering once inherent to large-incision surgery. Within a single generation, this once highly contested surgical innovation became the global standard of care, transforming surgical practice across disciplines and on a global scale. By every measure of public health, these outcomes place modern minimally invasive and robotic-assisted surgery as among the most consequential life-saving advances in modern medical history. This review examines the clinical impact and global dissemination of MIS, tracing its evolution from Camran Nezhat’s pioneering expansion of laparoscopy beyond diagnostics to complex therapeutic procedures across surgical disciplines. Drawing on decades of evidence across gynecology, general surgery, and urology, we show that MIS is associated with substantial reductions in perioperative mortality, major complications, blood loss, infections, thromboembolic events, postoperative pain, and length of hospital stay, while maintaining oncologic equivalence and improving functional and quality-of-life outcomes. Beyond these technical advances, MIS catalyzed a broader reimagining of surgery itself, challenging long-standing norms rooted in large-incision approaches and shifting the field toward precision, organ preservation, and pathology-directed intervention. These changes were accompanied by parallel advances in multiple domains, including in imaging, intraoperative visualization technologies, surgical anatomy, instrumentation, and nerve- and organ-sparing techniques—developments that collectively established the foundation for contemporary minimally invasive and robotic-assisted surgery. Collectively, these advances have contributed to the prevention of an estimated 10–20 million surgery-related deaths that would likely have occurred under the large-incision approaches of the past.

## 1. Introduction

Minimally invasive surgery (MIS) represents one of the most profound paradigm shifts in the history of modern medicine, fundamentally redefining surgical diagnosis and treatment across multiple specialties. Over the past century, MIS has evolved from rudimentary diagnostic endoscopy into a sophisticated, technology-driven operative platform encompassing many disciplines of surgery. This revolution reflects not only advances in instrumentation and imaging but also a broader philosophical transition toward patient-centered, tissue-sparing, and outcome-focused surgical care [1,2].

The conceptual origins of MIS date back to the late nineteenth and early twentieth centuries, when early endoscopic techniques were first explored. In 1901, Georg Kelling performed one of the earliest documented laparoscopic procedures in an animal model using a cystoscope, followed shortly thereafter by Hans Christian Jacobaeus, who applied laparoscopy to human patients for diagnostic evaluation of serous cavities [1,2]. Despite their novelty, these early techniques were constrained by limited optical quality, inadequate illumination, and significant safety concerns, restricting their use primarily to diagnostic purposes for several decades. A major turning point occurred in the mid-twentieth century with the introduction of the “cold light” fiber optic cable, safer trocar systems, and carbon dioxide pneumoperitoneum, which established the technical foundation for modern MIS [3].

Gynecology as a Pioneer: Gynecology played a foundational role in transforming MIS from a diagnostic curiosity into a therapeutic discipline. During the 1960s and 1970s, gynecologic innovators such as Kurt Semm advanced operative laparoscopy for procedures including tubal sterilization, ovarian surgery, and management of ectopic pregnancy. Semm’s landmark laparoscopic appendectomy in 1980—performed by a gynecologist—challenged traditional surgical hierarchies and underscored gynecology’s leadership in minimally invasive innovation [4]. Although initially met with skepticism, laparoscopy consistently demonstrated reduced postoperative pain, shorter hospitalization, faster recovery, and superior cosmetic outcomes. These advantages established gynecology as the proving ground for MIS and provided compelling evidence that minimally invasive approaches could safely replace open surgery at least for some pelvic pathologies [5].

Emergence of Video- Assisted MIS: A defining milestone in the MIS revolution was the introduction of video-assisted surgery, a paradigm shift invented and pioneered by Dr Camran Nezhat. In the late 1970s and early 1980s, Nezhat introduced the concept of operating while viewing the surgical field on a video monitor rather than directly through the eyepiece [6,7,8]. Prior to this, surgeons operated with limited ergonomics, constrained visualization, and minimal involvement of the surgical team. By integrating a video camera with the endoscope, Nezhat enabled simultaneous visualization for the entire operating room team, markedly improving precision, safety, ergonomics, documentation, and education. This innovation allowed surgeons to perform longer and more complex laparoscopic procedures, including advanced endometriosis excision, extensive adhesion lysis, bowel surgery, and ureteral dissection—procedures previously considered unsuitable for minimally invasive approaches [6,9].

Crucially, video-assisted MIS represented not merely a technological upgrade but a conceptual revolution in surgical practice. It enabled standardized training, objective peer review, procedural documentation, and global dissemination of advanced techniques [6,7,9,10]. This model of video-guided surgery directly influenced the subsequent adoption of MIS across many disciplines of surgery.

Across all specialties, MIS encountered early resistance related to safety concerns, training requirements, and perceived costs. However, consistent evidence demonstrating improved outcomes, combined with technological refinement and professional advocacy, transformed MIS from a controversial innovation into a surgical standard. Today, MIS is firmly embedded across many disciplines of surgery. From gynecology’s pioneering role to the transformative impact of video-assisted surgery, MIS has fundamentally reshaped modern surgical care. Its revolution serves as a reminder that meaningful progress often requires challenging convention, embracing innovation, and maintaining an unwavering focus on improving patient outcomes.

The following narrative is presented through the historical lens and professional perspective of Dr. Camran Nezhat, the central figure in the revolution of minimally invasive and video-assisted endoscopic surgery, for the purpose of posterity. This perspective aims to contextualize the revolution of MIS through the experience of those directly involved in shaping its course and advancing its impact on modern surgical care. In addition, this article reviews the achievements of MIS over a fifty-year period, from 1975 to 2025, across multiple surgical specialties, comparing MIS with open surgery with regard to mortality, morbidity, cost-effectiveness, surgeon preferences, and surgical volume.

## 2. The Revolution in Surgery That Saves Millions of Lives

The conceptual roots of MIS trace back to the eighteenth century, when Philipp Bozzini introduced the Lichtleiter, an early optical device designed to illuminate internal anatomical structures. While primitive by contemporary standards, this invention introduced a radical idea: that direct exposure of internal organs through large incisions was not an absolute prerequisite for surgical intervention. A small number of forward-thinking surgeons recognized that internal access through natural or created cavities could substantially reduce operative trauma and improve recovery, thereby laying the intellectual groundwork for what would later evolve into MIS.

Throughout the twentieth century, several innovators advanced this vision. Surgeons such as Raoul Palmer and Hubert Manhès in France, Kurt Semm in Germany, Victor Gomel in Canada, and George Berci, Harry Hasson, Harry Reich, David Redwine, James Daniell, and Dan Martin in the United States contributed critical technical and conceptual advances to endoscopic surgery. These pioneers demonstrated that operative intervention could be achieved through small access points, offering patients faster recovery and less postoperative morbidity compared with open procedures. However, despite these early successes, the broader clinical impact of endoscopy remained limited for decades.

**Figure 1:**
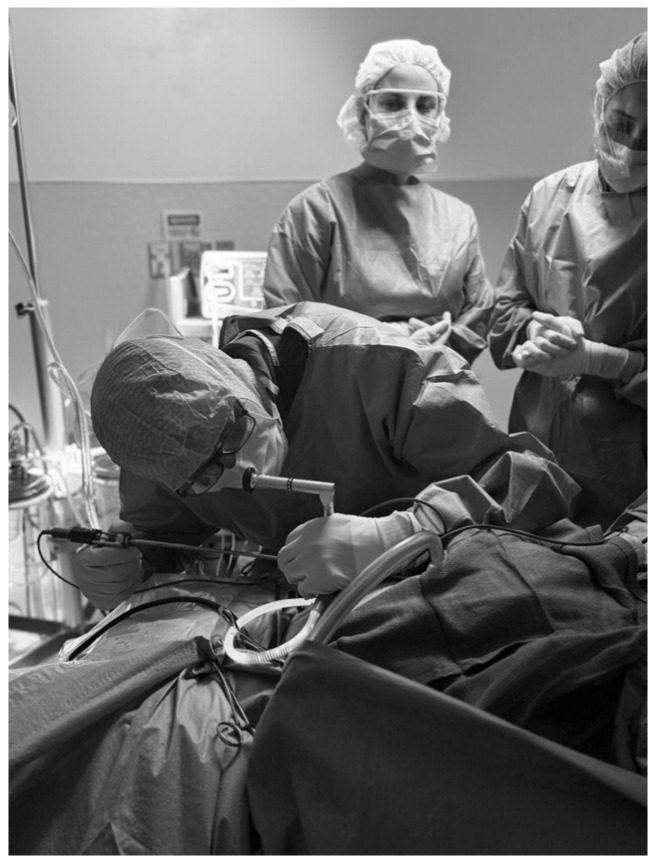
Depiction of awkward positioning necessary to use laparoscope. Images from the personal archive of Dr. Camran Nezhat; reproduced with permission.

A major barrier to widespread adoption was technological. Early laparoscopic procedures relied on monocular visualization through a rigid eyepiece, forcing surgeons into ergonomically unfavorable positions and constraining operative dexterity. Because one hand was required to stabilize the laparoscope, surgeons were often limited to single-handed operative maneuvers, increasing technical difficulty and limiting procedural complexity (Figure 1). Kurt Semm attempted to mitigate this limitation by securing the laparoscope with a head-mounted support system, allowing both hands to operate. While innovative, this approach proved difficult to master, challenging to teach, and unsuitable for routine clinical practice.

The turning point in the evolution of MIS occurred with a conceptual—not merely technological—advance: the transition from direct eyepiece viewing to complete reliance on video-based visualization. This shift enabled surgeons to perform entire operations while viewing a monitor rather than the operative field directly. Video-assisted laparoscopy fundamentally altered the mechanics of surgery, freeing both hands for precise bimanual control, improving ergonomics, and allowing the entire operating room team to share a common visual reference.

This approach was pioneered, invented and systematically developed by Dr. Camran Nezhat, who introduced the principle that operative performance could be safely and effectively conducted entirely from a video display. This innovation transformed surgery from an isolated, operator-dependent task into a coordinated, team-based clinical activity. Dr. Nezhat articulated a foundational concept that would later define the philosophy of MIS: **wherever a cavity exists within the body—or can be created—MIS is feasible and probably, preferable. The primary constraints, he emphasized, are the surgeon’s skill and experience and the availability of appropriate instrumentation**, a framework first formalized in publications from 1986 and 1990 [6,11,12].

At the time these ideas were introduced, they challenged prevailing surgical doctrine. In 1990, Dr. Nezhat predicted that most laparotomies would eventually be replaced by video-assisted techniques and that future surgeons might require specialized training to perform open surgery itself [12,13].

These assertions were met with skepticism and, in some cases, outright opposition. Prior to this work, no surgeon had completed full operative procedures while relying exclusively on video imaging displayed on a monitor. Although video technology existed in other contexts, it had not been applied to conduct entire surgical operations “off the screen”.

The proposal to abandon the eyepiece was widely criticized. Detractors questioned the safety, reproducibility, and teachability of operating via video display, characterizing the approach as unsafe or impractical. This skepticism extended even to respected contemporaries, including Drs. Dan Martin and Kurt Semm, and culminated in public debate at an international endoscopy meeting in Amsterdam in September 1988 [4]. Over time, however, many early critics adopted video-assisted techniques and later acknowledged their transformative impact, including formal endorsements in subsequent publications.

Despite resistance, the conviction underlying video-assisted laparoscopy remained unchanged: shared visualization enables safer, more precise, and more reproducible surgery. By liberating surgeons from restrictive optical systems and enabling coordinated teamwork, video-based surgery created the conditions necessary for complex procedures to be performed with reduced morbidity and improved outcomes. This conceptual shift would ultimately serve as the catalyst for the modern era of MIS.

Table 1 summarizes the landmark contributions of the major pioneers of minimally invasive surgery across gynecology, general surgery, urology, and thoracic—spanning over two centuries from Bozzini’s first optical device in 1806 to the advanced video-assisted procedures of the 1990s. Together, these innovators transformed surgery from an era of open incisions into one defined by precision, smaller wounds, and faster recovery.

### 2.1. Early Training and the Emergence of a Video-Based Surgical Paradigm

Dr. Camran Nezhat’s formal surgical training began in 1974 with an internship and residency in Obstetrics and Gynecology at the State University of New York at Buffalo, Millard Fillmore Hospital, under the mentorship of Dr. Ron Batt At that time, operative culture across most surgical disciplines favored extensive incisions and direct manual exposure, reflecting a long-standing belief that—**the larger the incision the bigger the surgeon!** Laparoscopy, although previously explored, had only cautiously re-entered clinical practice in the United States following a period of intense scrutiny and regulatory restriction related to complications from tubal ligation procedures. As a result, minimally invasive techniques were viewed with skepticism by much of the surgical community.

During this period, Dr. Nezhat demonstrated early technical aptitude in laparoscopic procedures but became increasingly aware of the limitations imposed by traditional optical systems. Reliance on a narrow eyepiece restricted situational awareness, constrained ergonomics, and isolated the surgeon from the rest of the operating team. The surgeon functioned largely alone, with limited visual input available to assistants, trainees, anesthesiologists, or nursing staff. This isolation not only impeded teaching but also limited procedural complexity and reproducibility.

These constraints prompted a fundamental question: could surgery be performed more safely and effectively if visualization were shared rather than individualized? Dr. Nezhat envisioned a surgical environment in which the laparoscopic image was projected onto a monitor, allowing the entire operating room team to observe the same operative field simultaneously. Such an arrangement would permit upright posture, true bimanual technique, and coordinated teamwork, transforming laparoscopy from a solitary technical exercise into a collaborative clinical process.

Between 1974 and 1980, during residency with Dr. Marvin Pleskow as chairman and subsequent fellowship training with Dr. Robert Greenblatt —an internationally recognized authority in endometriosis and infertility in Augusta, Georgia—Dr. Nezhat began systematically developing this concept. At the time, commercially available systems capable of supporting full video-guided surgery did not exist. To overcome this limitation, industrial cameras and later arthroscopic imaging systems were adapted for operative use, sometimes requiring the employment of a second laparoscope dedicated solely to illumination [57,58]. These early adaptations demanded significant technical improvisation but gradually demonstrated that stable, high-quality visualization could be achieved without direct eyepiece viewing.

As xenon and other cold-light sources became available, image clarity improved substantially, enabling increasingly complex procedures to be performed safely while relying exclusively on monitor-based visualization. These developments marked the first successful execution of hands-free, fully video-guided operative laparoscopy. Importantly, these procedures were not limited to diagnostic exploration but extended to therapeutic interventions, establishing the feasibility of conducting complete operations under video guidance alone.

Initial reactions from the broader surgical community were largely dismissive. Critics argued that operating from a monitor would compromise depth perception, increase procedural risk, and prove impossible to teach on a large scale. Some warned that the approach was inherently dangerous and should not be promoted beyond isolated experimentation. Despite these objections, continued refinement of technique, combined with consistent clinical success, provided mounting evidence that video-assisted surgery was not only feasible but advantageous.

**Figure 2:**
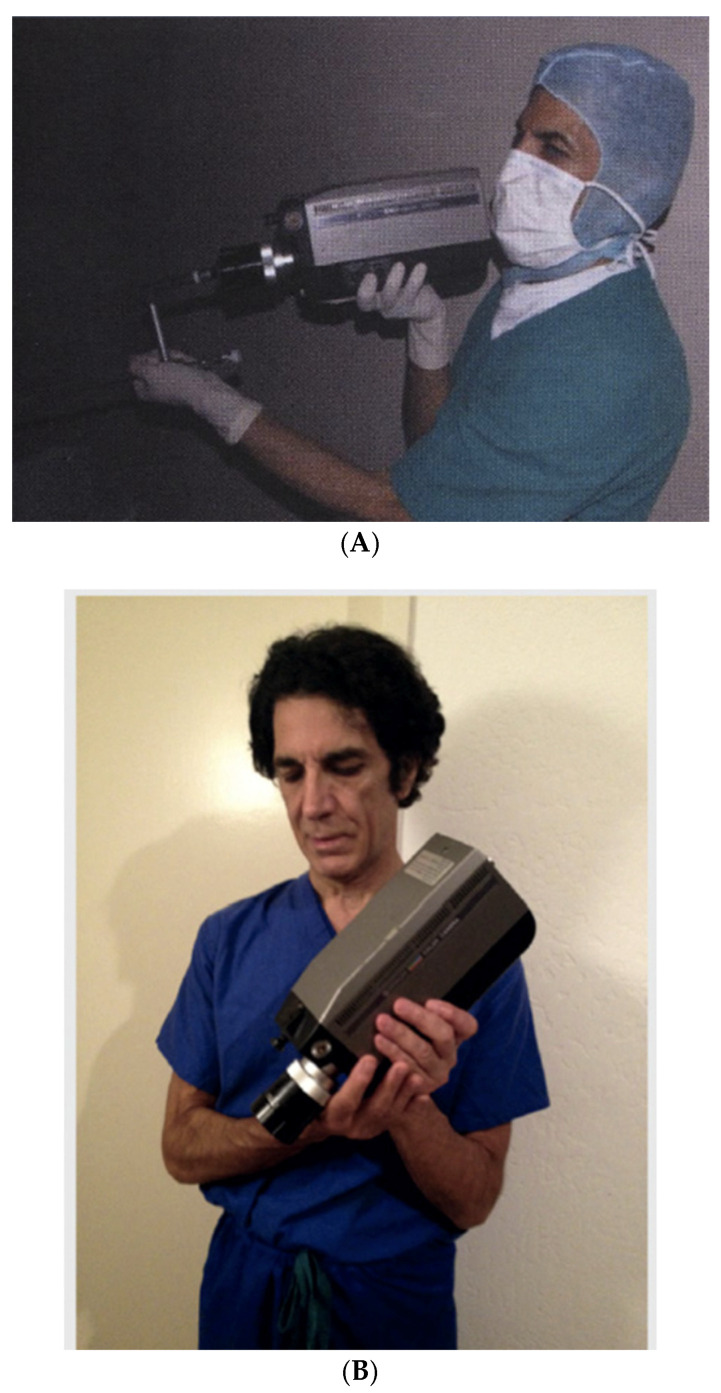
(**A**,**B**) Dr. Camran Nezhat with bulky camera in the late 1970s and early 1980s. Images from the personal archive of Dr. Camran Nezhat.

**Figure 3:**
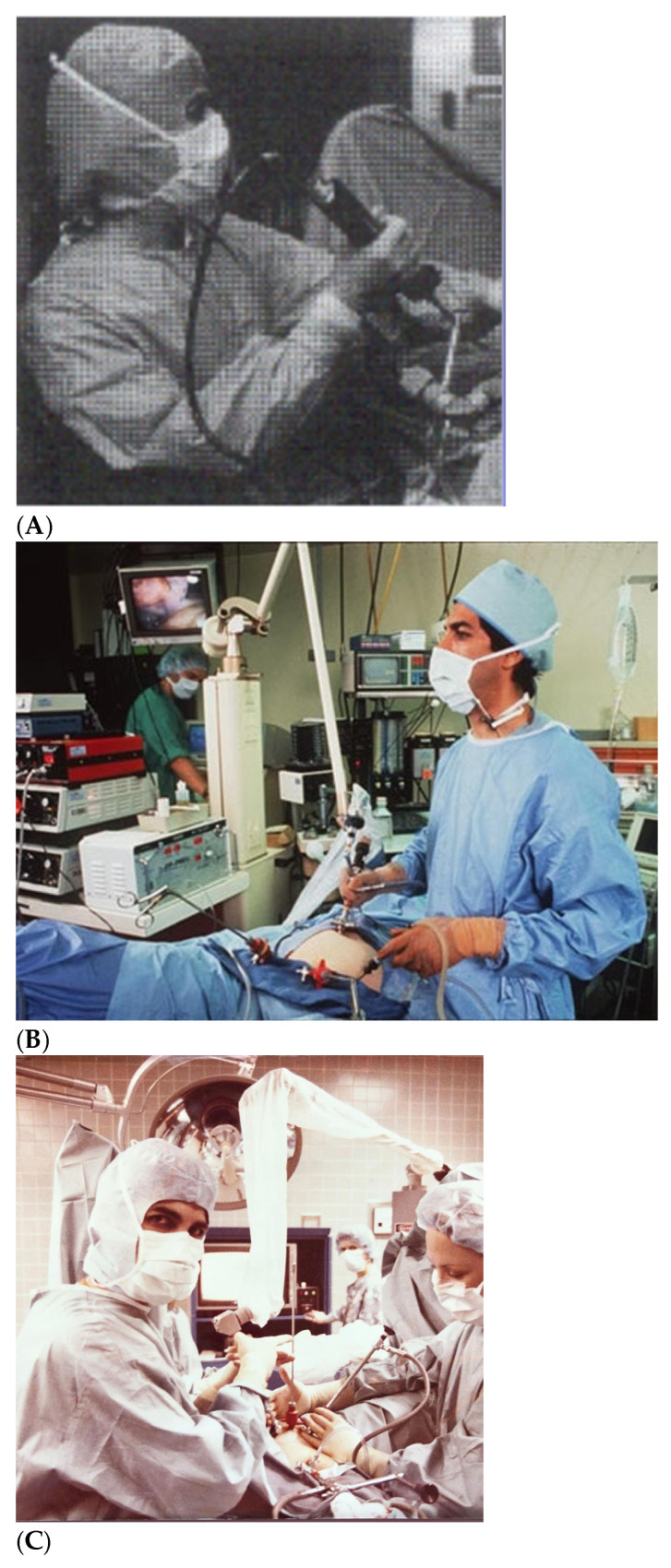
(**A**). Camran Nezhat in the early 1980s. (**B**). Videolaparoscopy with video monitor in the mid 1980s. Not many accessories were available, so, for example, Dr. Nezhat bought an insufflator from Germany and brought it to the United States. (**C**). Early videolaparoscopy in the late 70s and early 80s. Due to the lack of adequate light and the poor resolution of the camera, we used two laparo-scopes in the abdomen to deliver more light to perform the procedures. Images from the personal archive of Dr. Camran Nezhat.

Through collaboration with engineers and continued technical iteration, one of the first fully functional video-laparoscopic systems was developed, fundamentally altering operating room dynamics (Figure 2 and Figure 3). Surgeons were no longer tethered to optical eyepieces; instead, they could operate with both hands while maintaining constant visual awareness of the operative field. Equally important, assistants, trainees, and nursing staff could now anticipate operative steps, respond more effectively to intraoperative events, and participate meaningfully in the surgical process.

The implications of this transformation extended beyond ergonomics. Video-assisted laparoscopy introduced a new level of procedural standardization and transparency. Surgical steps could be recorded, reviewed, taught, and disseminated, facilitating quality control and accelerating the spread of technical expertise. What emerged was not simply a new tool, but a new surgical paradigm—one in which visualization, teamwork, and reproducibility became central determinants of clinical success.

This foundational phase established the conditions necessary for the broader clinical expansion of MIS. By demonstrating that complex operative tasks could be performed safely under exclusive video guidance, video-assisted laparoscopy laid the groundwork for its subsequent application across a wide range of pathologies and surgical specialties.

### 2.2. Clinical Implementation and Early Demonstration of Efficacy

Following completion of fellowship training in 1980, Dr. Camran Nezhat established his clinical practice in Atlanta, Georgia, with a primary focus on infertility and endometriosis. This period marked the transition from experimental development to real-world clinical application of video-assisted MIS. At the time, treatment options for advanced endometriosis were largely limited to laparotomy, frequently associated with prolonged recovery, significant postoperative pain, and high morbidity.

Early application of video-assisted laparoscopy to the management of endometriosis produced outcomes that contrasted sharply with prevailing expectations. Patients experienced reduced postoperative discomfort, faster mobilization, and earlier return to daily activities. These clinical benefits quickly became apparent not only to patients themselves but also to referring physicians, who began directing increasingly complex cases to Dr. Nezhat’s practice.

The rapid dissemination of these outcomes occurred largely through patient experience and professional observation rather than formal academic endorsement.

As clinical volume increased, the scope of pathology treated using video-assisted techniques expanded. Procedures progressed from relatively limited interventions to extensive operations involving deeply infiltrative and multiorgan disease. Working with a dedicated multidisciplinary team, Dr. Nezhat demonstrated that even stage IV endometriosis affecting the bowel, bladder, ureters, diaphragm, and other structures could be safely managed laparoscopically [6,10,11,23,25,28,39,46,59,60,61,62,63,64,65,66,67,68].

These achievements challenged the assumption that MIS was suitable only for minor or superficial disease.

The physiological environment created by pneumoperitoneum further contributed to operative safety. Elevated intra-abdominal pressure reduced venous oozing from small vessels, improving visualization and decreasing blood loss during dissection. When combined with magnified video imaging, this environment enabled surgeons to identify anatomical planes and pathological involvement with a level of clarity not achievable during open surgery. As a result, surgical precision improved while tissue trauma was minimized.

During this period, video-assisted laparoscopy was also applied to procedures that had previously been performed primarily in Europe but not yet adopted in the United States. Among these was laparoscopic appendectomy, which was performed using video guidance for the first time domestically [32]. Introduction of these procedures further demonstrated the versatility of the technique and its applicability beyond gynecologic surgery. By extending minimally invasive approaches to commonly performed operations, a pathway was created for broader adoption across surgical disciplines.

An additional factor accelerating acceptance was systematic documentation of operative procedures. Surgeries were routinely recorded and shared with referring physicians and patients, providing transparent evidence of technique, outcomes, and safety. This practice not only enhanced patient understanding and confidence but also facilitated peer-to-peer learning at a time when formal training pathways for advanced laparoscopy were still limited.

As interest in video-assisted surgery grew, structured teaching efforts began. Dr. Nezhat initially collaborated with Dr. Scott Crowgey to provide instruction at the Georgia Institute of Technology and later at Northside Hospital. These early educational initiatives demonstrated that video-assisted laparoscopy was not restricted to individual technical virtuosity but could be taught systematically. The presence of a shared video display allowed learners to observe fine technical details in real time, ask questions during procedures, and develop a conceptual understanding of operative strategy.

Importantly, the transition from eyepiece-based to video-guided surgery altered the social dynamics of the operating room. Surgeons could maintain upright posture, operate with both hands, and coordinate seamlessly with assistants, nurses, and trainees who now shared the same visual information (Figure 3). Surgery evolved from an isolated technical act into a coordinated team effort, enhancing efficiency and intraoperative communication.

Through these early clinical successes, video-assisted laparoscopy demonstrated that MIS was not merely an alternative technique but a superior clinical strategy for reducing operative morbidity while maintaining—or improving—therapeutic effectiveness. These findings laid the foundation for subsequent multidisciplinary collaboration and expansion into additional surgical specialties.

### 2.3. Multidisciplinary Expansion and System-Level Dissemination

As clinical experience with video-assisted laparoscopy increased, its application extended beyond gynecology into multiple surgical disciplines. This expansion was driven not by theoretical promise alone, but by reproducible clinical results demonstrating that complex procedures could be performed with reduced morbidity and improved recovery. During the 1980–1990 period in Atlanta, video-assisted techniques were increasingly adopted in collaboration with surgeons from colorectal, urologic, thoracic, general, and oncologic specialties.

Close collaboration with colorectal surgeons—including Drs. Earl Pennington, Wayne Ambroze, Guy Orangio, and Marion Schertzer—enabled the laparoscopic management of advanced bowel pathology, including deeply infiltrative disease involving the rectosigmoid colon [10,39,63,64,65,66]. These procedures, once considered feasible only through open surgery, demonstrated that video-assisted laparoscopy could achieve precise dissection, safe resection, and effective reconstruction in anatomically complex regions.

Parallel advances occurred through collaboration with urologists Drs. Howard Rottenberg, Bruce Green, and Fred Shessel, who applied minimally invasive techniques to ureteral and bladder pathology [28,46]. Thoracic applications were developed with Dr. Howard Brown, while general surgical procedures were advanced in collaboration with Dr. John Harvey. In gynecologic oncology, joint efforts with Drs. Benedict Benigno, Matthew Burrell, and Charles Welander extended video-assisted approaches to oncologic resections, including radical procedures previously considered incompatible with minimally invasive techniques [23,67].

These multidisciplinary collaborations were not incidental; they were essential to the maturation of MIS as a health-system intervention. By working across specialties, operative principles could be adapted, refined, and standardized, accelerating adoption and improving consistency of care. Video-based visualization served as a unifying platform, allowing surgeons from different disciplines to share techniques, anticipate operative steps, and learn from one another in real time.

As outcomes continued to improve, public awareness of video-assisted surgery increased. Major media outlets—including Time, Newsweek, and 20/20—featured reports highlighting the clinical benefits of minimally invasive approaches. Notably, widespread acceptance was driven primarily by patient experience rather than institutional endorsement. Patients recognized the advantages of reduced pain, faster recovery, and shorter hospitalization, and this demand exerted pressure on health systems to adopt minimally invasive techniques [69,70,71].

**Figure 4:**
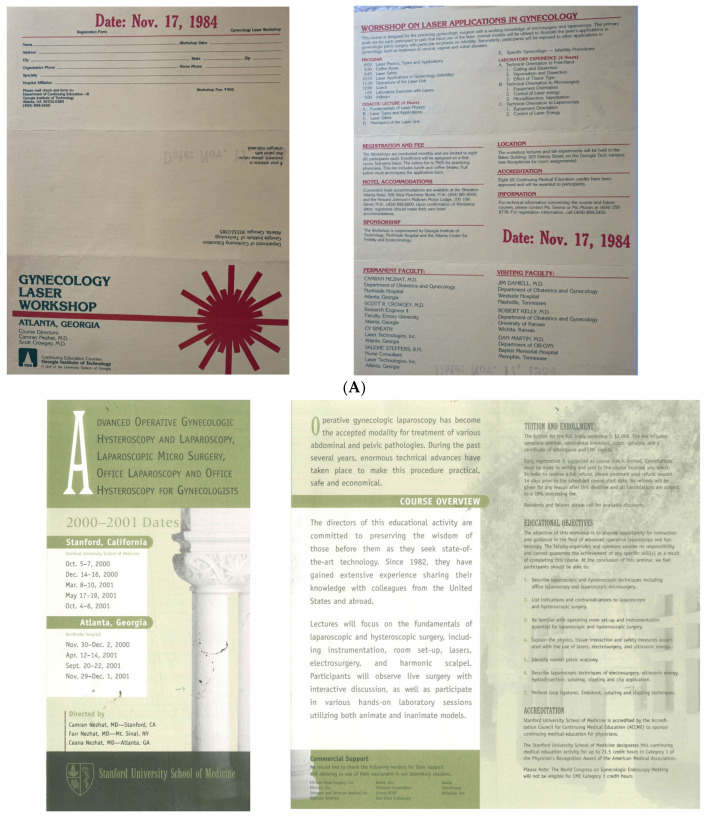

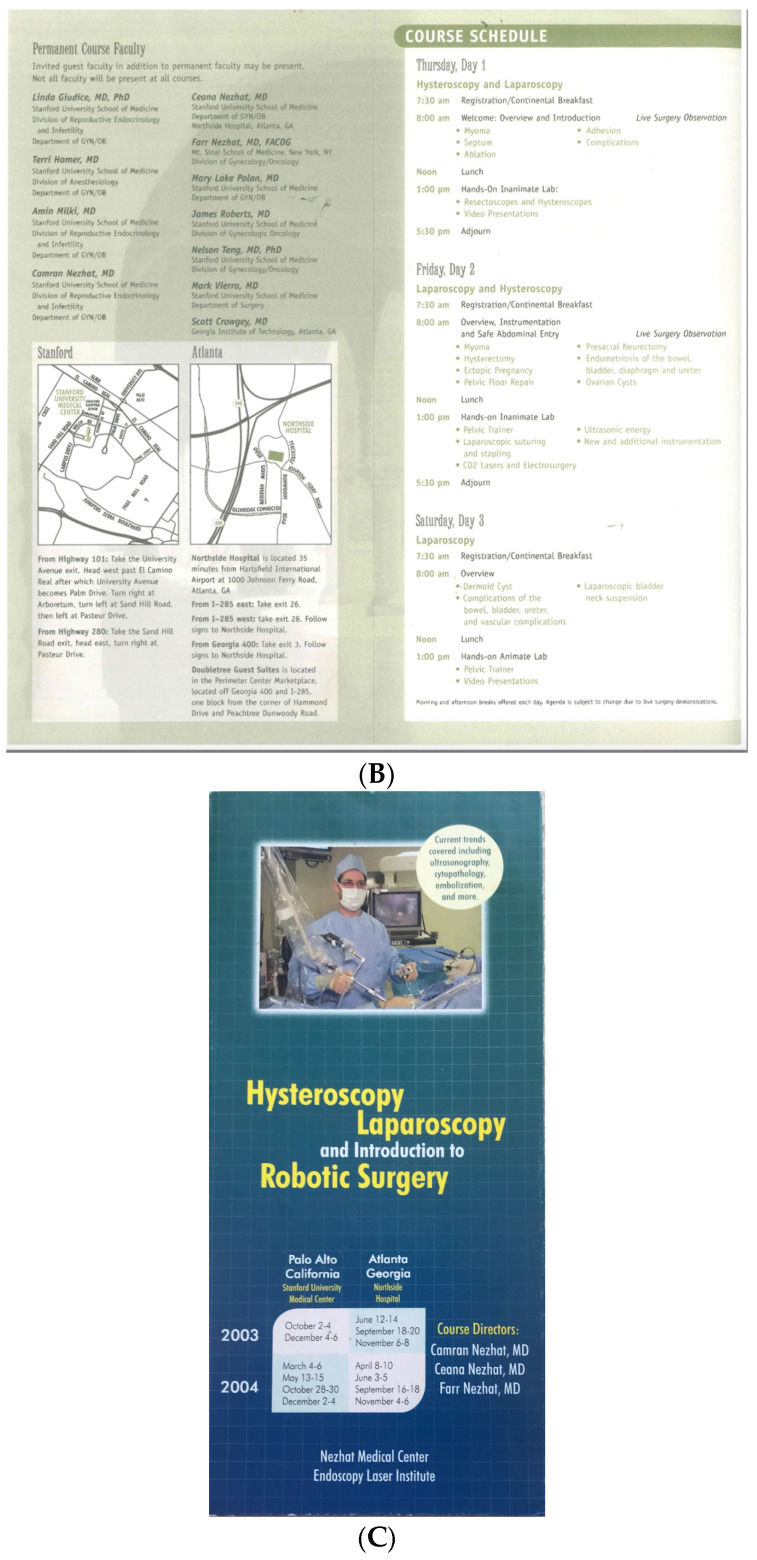
(**A**). Brochure for Gynecology Laser Workshop taught by Camran Nezhat and Scott Crowgey at Northside Hospital in November 1984. (**B**). Brochure for Advanced Operative Gyne-cologic Hysteroscopy and Laparoscopy, Laparoscopic Microsurgery, Office Laparoscopy and Office Hysteroscopy for Gynecologists in 2000 and 2001 at Northside Hospital in Atlanta, Georgia and Stanford University Medical Center in Palo Alto, California. (**C**). Brochure from Hysteroscopy, Laparoscopy, and Introduction to Robotic Surgery Postgraduate Course taught by Drs. Camran, Farr, and Ceana Nezhat in 2003 and 2004 at Northside Hospital in Atlanta, Georgia, and Stanford University Medical Center in Palo Alto, California. Images from the personal archive of Dr. Camran Nezhat.

During this period, Dr. Camran Nezhat worked closely with his brothers, Dr. Farr Nezhat and Dr. Ceana Nezhat, both of whom played integral roles in refining operative techniques and expanding educational efforts. Together, they established structured postgraduate training programs that evolved from occasional courses to nearly monthly sessions (Figure 4). Surgeons from across the United States and internationally—including gynecologists, urologists, and general surgeons—visited these operating rooms to observe procedures and gain firsthand experience with video-assisted techniques.

The educational impact of these efforts extended beyond individual skill acquisition. Video-assisted surgery enabled direct visualization of operative decision-making, facilitating a deeper understanding of anatomy, pathology, and surgical strategy. Procedures could be recorded, reviewed, and disseminated, creating an early framework for quality assurance and continuous improvement. These features transformed surgical education from an apprenticeship model reliant on limited observation to a scalable system capable of training large numbers of surgeons with consistent standards.

Despite increasing clinical adoption and patient demand, resistance within academic institutions persisted. Some academic leaders dismissed video-assisted surgery as a transient trend rather than a durable improvement in care. However, the accumulating body of clinical evidence and the rapid expansion across specialties underscored a critical reality: MIS was not confined to a single discipline or disease category but represented a broadly applicable strategy for reducing surgical harm while preserving therapeutic efficacy.

By the end of the 1980s, video-assisted laparoscopy had moved beyond early experimentation. It had become a clinically validated, teachable, and scalable approach capable of transforming operative practice across health systems. This momentum set the stage for broader academic engagement, international dissemination, and eventual integration into standard surgical training and care pathways.

### 2.4. Academic Resistance, Early Validation, and International Dissemination

Despite growing clinical success and increasing patient demand, the integration of video-assisted laparoscopy into mainstream academic medicine progressed unevenly. Many academic institutions and professional societies initially viewed the technique with caution, questioning its safety, reproducibility, and long-term outcomes. This skepticism was not uncommon for disruptive medical technologies that challenged established norms and threatened existing hierarchies within surgical practice.

A small number of academic editors, however, recognized the scientific merit and clinical implications of video-assisted MIS. Visionary leadership from editors such as Dr. Alan DeCherney at Fertility and Sterility and Dr. Karl Zucker at Surgical Laparoscopy, Endoscopy & Percutaneous Techniques enabled early peer-reviewed publication of foundational work in video-assisted surgery. These publications were instrumental in legitimizing the technique within academic discourse and provided an evidence base for further investigation and adoption.

During this period, external pressures emerged that tested the resilience of academic publishing. A lawyer—later sanctioned by the courts—initiated legal threats against journals and attempted to influence editorial decisions by demanding retraction of published work. While most editors upheld the integrity of peer review and resisted intimidation, the sudden death of Dr. Karl Zucker led to a change in editorial leadership at one journal. Subsequently, two previously published articles were withdrawn without author notification or scientific reassessment, despite the fact that the work had already undergone independent academic review and validation at Stanford University Medical Center.

Repeated efforts to republish the withdrawn material were unsuccessful. Even formal inquiries from Dr. Michael Kavic, Editor of the Journal of the Society of Laparoscopic and Robotic Surgeons (JSLS), requesting permission to reprint the articles received no response. These events underscored the vulnerability of innovation during periods of institutional transition and highlighted the importance of editorial independence in advancing evidence-based medicine.

Notwithstanding these obstacles, clinical investigation continued uninterrupted. In the mid-1980s, peer-reviewed studies documented successful video-assisted laparoscopic management of mild to severe endometriosis, including extensive multiorgan disease, with favorable outcomes and preserved fertility [6,11].

These findings supported a broader clinical inference: if advanced stage IV endometriosis involving multiple organ systems could be managed laparoscopically, then the boundaries of MIS were far wider than previously assumed. This conclusion, initially regarded as provocative, would later prove prescient.

A parallel principle emerged from this work—that minimally invasive surgery is appropriate wherever a cavity exists within the body or can be safely created, provided sufficient surgical expertise and appropriate instrumentation are available [6]. This concept offered a unifying framework for extending video-assisted techniques across surgical specialties and disease categories.

International engagement played a pivotal role in accelerating dissemination. In 1985, Dr. Camran Nezhat was invited by Professor Maurice Bruhat to Clermont-Ferrand, France, to demonstrate video-assisted endoscopic surgery. During this visit, he interacted with members of Professor Bruhat’s department, including Professors Hubert Manhès, Gérard Mage, Jean-Luc Pouly, Michel Canis, and Arnaud Wattiez, many of whom continued to advance minimally invasive techniques in Europe.

**Figure 5:**
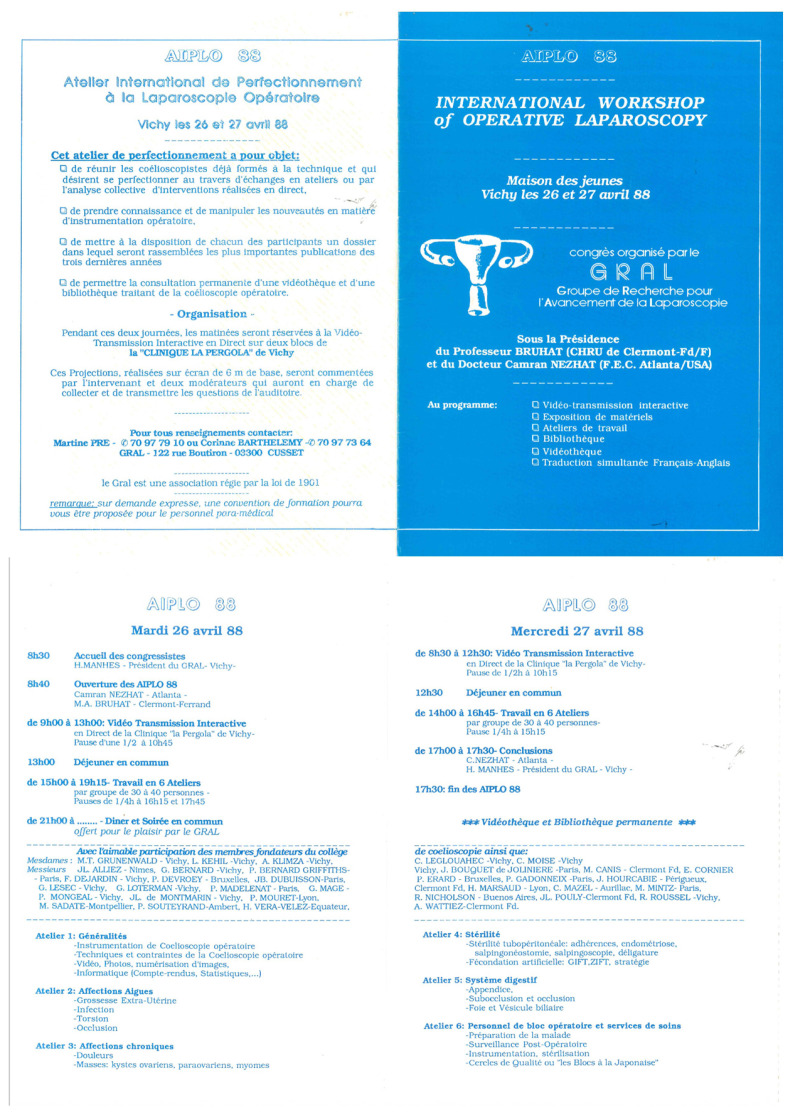
Program from the International Workshop of Operative Laparoscopy held in Vichy, France, 26–27 April 1988. The original program was in French; key sections include registration, lectures, video demonstrations, hands-on workshops, and concluding sessions. Images from the personal archive of Dr. Camran Nezhat.

At the same meeting, Dr. Nezhat met Dr. Philippe Mouret, a general surgeon with experience in gynecologic procedures Over subsequent years, Dr. Nezhat returned to France multiple times to participate in postgraduate educational programs. In April 1988, he served as co-director of a postgraduate course in Vichy with Professor Bruhat, held in honor of Professor Manhès (Figure 5).

By that time, Dr. Mouret had begun performing video-assisted laparoscopic cholecystectomy, initiating the procedure in 1987. Following his presentation in Vichy, Dr. Mouret shared video recordings of these operations with attendees, including Dr. Nezhat and several American surgeons such as Dr. Harry Reich, Dr. James Daniell, and Dr. Donald Chatman. These recordings provided compelling visual evidence that complex general surgical procedures could be performed safely using video-assisted techniques.

Upon returning to Atlanta, Dr. Nezhat presented the cholecystectomy videos during postgraduate courses at Northside Hospital, generating immediate interest among general surgeons. Although institutional approval was initially denied, collaboration between Dr. William Saye, a gynecologist, and Dr. William McKernan, a general surgeon, led to the first laparoscopic cholecystectomy performed in the United States in May 1988 at Dr. Saye’s outpatient surgery center.

Other surgeons who attended the Vichy meeting pursued similar efforts upon returning to their institutions. Dr. James Daniell subsequently collaborated with Dr. Eddie Joe Reddick, who performed a laparoscopic cholecystectomy in Nashville, Tennessee, in June 1988. Later that year, Dr. Nezhat worked with Dr. John Harvey to perform video-assisted cholecystectomy in Atlanta.

Once general surgeons observed the clinical advantages of laparoscopic cholecystectomy—including smaller incisions, reduced postoperative pain, and faster recovery—the pace of adoption accelerated rapidly. The procedure spread in MIS minimally invasive surgery across surgical disciplines.

### 2.5. Institutional Adoption, Professional Societies, and Academic

As MIS gained clinical momentum, professional organizations played a critical role in formalizing its adoption and dissemination. Among these, the Society of American Gastrointestinal and Endoscopic Surgeons (SAGES) emerged as a key advocate for laparoscopic techniques. By embracing video-assisted surgery early and developing structured educational initiatives such as the Fundamentals of Laparoscopic Surgery (FLS) program, SAGES helped establish standardized benchmarks for training, credentialing, and patient safety. These efforts were instrumental in transitioning MIS from an emerging technique into an accepted standard of care.

With accumulating evidence supporting the safety and efficacy of video-assisted procedures, even traditionally conservative academic institutions began to reconsider their positions. Invitations to demonstrate video-assisted surgery at major academic medical centers increased, signaling a broader shift in institutional attitudes. One such milestone occurred when Dr. Camran Nezhat was invited by Dr. John Lewis, Chair of the Department of Gynecologic Surgery, and Dr. Murray Brennan, Chair of the Department of Surgery, to present and demonstrate advanced video-assisted procedures at Memorial Sloan Kettering Cancer Center in New York.

During this visit, Dr. Nezhat performed video-assisted laparoscopic radical hysterectomy with para-aortic and pelvic lymph node dissection, as well as video-assisted ileocecectomy, procedures that were traditionally performed through open approaches. The favorable reception of these demonstrations underscored the growing recognition that MIS could be applied safely to complex oncologic and gastrointestinal operations in high-acuity academic settings.

The impact of the visit was reflected in letters from two of Memorial Sloan-Kettering’s most senior figures. Dr. John I. Lewis, Jr., wrote on 1 June 1992, to express his admiration for Dr. Nezhat’s contributions, stating that his “ability to outline the rationale and techniques of videoscopically-controlled laparascopic surgery is outstanding and your technical skills speak for themselves.” Equally telling was the response of Dr. Murray F. Brennan, Chairman of the Department of Surgery and holder of the Alfred P. Sloan Chair in Surgery, who wrote on 1 July 1992, that he “very much enjoyed your visit” and moved swiftly to propose sending one of his young attending staff to Atlanta, along with a nurse and technician, to assess equipment needs and observe Dr. Nezhat’s techniques firsthand.

A defining phase in the institutional integration of video-assisted MIS began with collaboration between Dr. Nezhat and Stanford University Medical Center. This partnership originated when Mr. Kenneth Bloom, Chief Executive Officer of Stanford Medical Center, and Ms. Linda Meier, then Chair of the Board, observed video-assisted surgery firsthand in Atlanta. Impressed by the precision, teamwork, and clinical efficiency enabled by shared visualization, they encouraged the development of a comprehensive MIS program at Stanford.

After several years of planning and discussion, Dr. Nezhat relocated to Stanford University Medical Center in 1993, where he continued to advance clinical practice, education, and interdisciplinary collaboration. Initial skepticism within the institution mirrored that seen elsewhere. Dr. John Niederhuber, then Chair of the Department of Surgery and a surgical oncologist, initially questioned the applicability of video-assisted approaches to complex abdominal procedures.

This skepticism shifted following direct observation of a laparoscopic bowel resection performed by Drs. Camran Nezhat and Mark Vierra. Recognizing the clinical advantages of the approach, Dr. Niederhuber not only adopted the technique personally but also supported its broader integration within the department, ultimately appointing Dr. Nezhat to a professorship in the Department of Surgery.

Under the leadership of senior administrators and academic leaders—including Dr. David K. Stevenson (Vice Dean and Senior Associate Dean), Dr. Lloyd Minor (Dean), and former Deans Drs. Eugene Bauer and Richard Popp—Stanford University Medical Center became one of the earliest institutions to establish a comprehensive center dedicated to MIS. This institutional commitment facilitated cross-disciplinary collaboration and accelerated the integration of video-assisted techniques into routine clinical care.

Support from departmental leadership further reinforced this transformation. Dr. Mary Hawn, Chair of the Department of Surgery; Drs. Christopher Zarins and Thomas Krummel, former Chairs of Surgery; Dr. Mary Lake Polan, former Chair of Obstetrics and Gynecology; and Dr. Carl Levinson, former Director of Stanford Endoscopy for Training and Technology, all played key roles in sustaining and expanding minimally invasive surgical programs.

At Stanford, collaboration extended across a wide range of surgical disciplines. Dr. Nezhat worked closely with colorectal, urologic, thoracic, cardiac, and neurosurgical colleagues, including Drs. Mark Welton, Andrew Shelton, Cindy Kin, Natale Kirilcuk, Fuad Frieha, Christopher Payne, Harcharan Gill, Benjamin Chung, Ramin Beygui, Leah Backhus, and John Adler. These partnerships demonstrated that video-assisted surgery was not confined to a single specialty but represented a versatile platform adaptable to diverse clinical contexts.

Through institutional endorsement, standardized training, and interdisciplinary collaboration, video-assisted MIS transitioned from an innovative technique into an integrated component of academic surgical practice. This phase marked a critical step toward the widespread normalization of minimally invasive approaches within modern health systems.

By 1990, Nezhat and colleagues had conclusively demonstrated that even the most advanced and complex pathologies could be safely managed using video-assisted laparoscopy, with their findings disseminated by forward-thinking editors. Minimally invasive techniques were no longer confined to minor procedures but were shown to have the potential to transform the full spectrum of surgical practice. This body of work reinforced a principle articulated by Nezhat in 1986 and 1990: wherever a cavity exists or can be created within the body, MIS is feasible and often preferable. The primary limiting factors are the surgeon’s skill and experience, along with the availability of appropriate instrumentation [6,10,11,20,22,23,24,25,26,27,28,29,39,46,47,48,53,54,55,56,59,60,61,62,63,64,65,66,67,68,71,72,73,74,75,76,77,78,79,80,81].

### 2.6. Industry Collaboration and Technological Advancement

The clinical expansion of video-assisted MIS was closely linked to parallel advances in surgical technology. As operative complexity increased, collaboration between surgeons and industry leaders became essential to translate conceptual innovation into reliable clinical tools. During the mid- to late-1980s, several individuals and companies played pivotal roles in developing instrumentation that enabled complex laparoscopic procedures to be performed safely and reproducibly.

Leon Hirsch of U.S. Surgical was among the earliest industry partners to recognize the transformative potential of video-assisted surgery. His team developed advanced stapling devices, including vascular staplers, which allowed secure hemostasis and tissue division during laparoscopic resections. The clinical utility of these devices was demonstrated in early reports describing their application in laparoscopic hysterectomy [82]. Notably, the first laparoscopic hysterectomy had been performed by Dr. Harry Reich using radiofrequency bipolar energy to achieve vascular control, underscoring the rapid evolution of hemostatic technologies in MIS.

In parallel, William Weldon of Johnson & Johnson supported the development of safer and more reliable access systems, addressing one of the early technical challenges of laparoscopy—secure entry into the abdominal cavity. Improvements in trocar design and access techniques contributed significantly to reducing entry-related complications and increasing surgeon confidence in minimally invasive approaches.

Advances in imaging technology were equally critical. Richard Auhll of Circon facilitated the development of durable, high-resolution video camera systems capable of delivering stable, high-quality images throughout prolonged procedures. These imaging platforms enabled surgeons to maintain consistent visualization during complex dissections, further enhancing safety and precision.

Optical innovation was also advanced through the longstanding contributions of Karl and Sybill Storz and the Karl Storz Company, whose commitment to optical excellence supported the refinement of laparoscopic lenses, light transmission, and visualization systems. These developments collectively transformed video-assisted laparoscopy from a technically demanding innovation into a dependable clinical modality.

As video-assisted surgery matured, attention turned toward further enhancing surgeon dexterity, ergonomics, and precision. In collaboration with Dr. Ajit Shah and Phil Green at the Stanford Research Institute, Dr. Nezhat participated in early development efforts that contributed to the creation of the da Vinci robotic surgical system, later commercialized by Intuitive Surgical in 1995. Robotic platforms extended the principles of video-assisted surgery by providing enhanced instrument articulation, tremor filtration, and improved ergonomics.

The introduction of robotic technology facilitated the broader dissemination of minimally invasive techniques, enabling surgeons with varying levels of experience to perform complex procedures across multiple disciplines and geographic regions. Robotics-assisted MIS did not replace the foundational concepts of video-assisted surgery but rather built upon them, reinforcing the central role of shared visualization and precise instrument control.

Academic dissemination of these advances was supported by editors and publishers who recognized the clinical importance of emerging technologies. Leaders such as Dr. Alan DeCherney (Fertility and Sterility), Dr. Michael Kavic (Journal of the Society of Laparoscopic and Robotic Surgery), and Dr. Paul Wetter, CEO of the Society of Laparoscopic and Robotic Surgeons (SLS), facilitated publication of pioneering work describing robotic-assisted resections and reconstructions involving the bowel, bladder, ureter, and other organs [9,13,29,54,73,78,79,80,81,83,84,85,86,87].

These publications documented that robotic-assisted surgery, when grounded in the principles of video-based visualization and meticulous technique, could achieve outcomes comparable to—and in some cases surpassing—traditional open approaches. Together, surgeon–industry collaboration and iterative technological refinement ensured that MIS continued to evolve in step with clinical demands, expanding its reach and impact within modern health systems.

### 2.7. Challenges, Oversight, and Institutional Validation

The introduction of transformative clinical innovations is frequently accompanied by resistance, particularly when established practices are disrupted. Video-assisted MIS was no exception. As its adoption accelerated and outcomes continued to improve, some critics characterized laparoscopy as experimental, unsafe, or transient, using terms such as “gimmick,” “foreverscopy,” or a temporary trend destined to fail [30,31,32,33]. In certain instances, opposition extended beyond scientific debate and entered the legal and media arenas.

A particularly aggressive legal campaign sought to undermine confidence in video-assisted surgery by advancing unsubstantiated allegations and exerting pressure on academic institutions, journals, and professional colleagues. These actions prompted formal investigations by multiple oversight bodies, reflecting the seriousness with which academic medicine evaluates claims related to patient safety and research integrity.

Stanford University Medical Center undertook three independent institutional investigations, each led by senior academic leadership and conducted with external oversight. These reviews were overseen at different times by Dr. Eugene Bauer, Dr. Richard Popp, and Dr. Philip Pizzo, and included evaluation by independent committees composed of respected clinicians, legal experts, and ethicists. In each instance, the investigations concluded that the clinical work, research practices, and educational activities associated with video-assisted MIS met accepted scientific and ethical standards.

One review, conducted by a blue-ribbon panel that included a former Dean of Medicine from Harvard University, a retired California Supreme Court Justice, and an ethicist, offered particularly strong conclusions. The panel determined that the allegations lacked merit and emphasized that future accusations should be evaluated in light of the credibility and motivations of accusers rather than presumption of wrongdoing.

In addition to institutional review, the work was scrutinized by multiple external agencies, including the Department of Justice, the Internal Revenue Service, the Federal Bureau of Investigation, and state medical boards. Each inquiry independently affirmed the absence of misconduct and validated the integrity of the clinical and research programs under review.

Judicial evaluation further reinforced these conclusions. The Eleventh Circuit Court of Appeals in Atlanta ruled decisively in favor of Dr. Nezhat and his colleagues, explicitly stating that they had been subjected to inappropriate treatment by legal and media entities and affirming their professional conduct [88]. These findings underscored the importance of due process and evidence-based assessment when evaluating disruptive medical innovations.

Throughout this period, continued clinical practice and research were sustained by the support of academic leaders who recognized the broader implications of video-assisted surgery for patient care. Figures such as Dr. David K. Stevenson provided steadfast institutional backing, ensuring that scientific inquiry and patient-centered innovation could proceed without compromise.

From a systems perspective, these events highlighted the necessity of robust governance mechanisms in the evaluation of new surgical technologies. Rigorous oversight, transparent investigation, and independent validation serve as essential safeguards—not only for patient safety, but also for protecting responsible innovation from suppression driven by misinformation or resistance to change. The eventual vindication of video-assisted MIS reinforced its legitimacy as a clinically superior approach grounded in reproducible outcomes and ethical practice.

### 2.8. Recognition, Population-Level Impact, and Transition to Standard of Care

By the early 2000s, the clinical value of video-assisted MIS had become widely acknowledged across medical disciplines. Techniques that were once viewed with skepticism were increasingly adopted as preferred approaches for a broad range of procedures, reflecting a shift from experimental application to routine clinical practice. This transition was driven not by novelty, but by accumulating evidence demonstrating consistent reductions in morbidity, faster recovery, and improved patient experience.

Recognition by leading medical journals further signaled this transition. In 2004, publications in the New England Journal of Medicine highlighted the superiority of minimally invasive [89,90]. At this point, video-assisted and minimally invasive techniques were no longer confined to early adopters; they had become embedded within surgical training programs and institutional standards worldwide.

The normalization of MIS altered expectations for operative care. Health systems increasingly regarded smaller incisions, reduced postoperative pain, and shorter hospital stays not as optional benefits but as benchmarks of quality. As a result, virtually all major academic centers and hospitals began to develop programs emphasizing minimally invasive and, later, robotic-assisted MIS. This widespread adoption represented a collective achievement in patient-centered care, even as the historical path to acceptance reflected the familiar pattern in which innovations are initially resisted before becoming mainstream.

**Figure 6:**
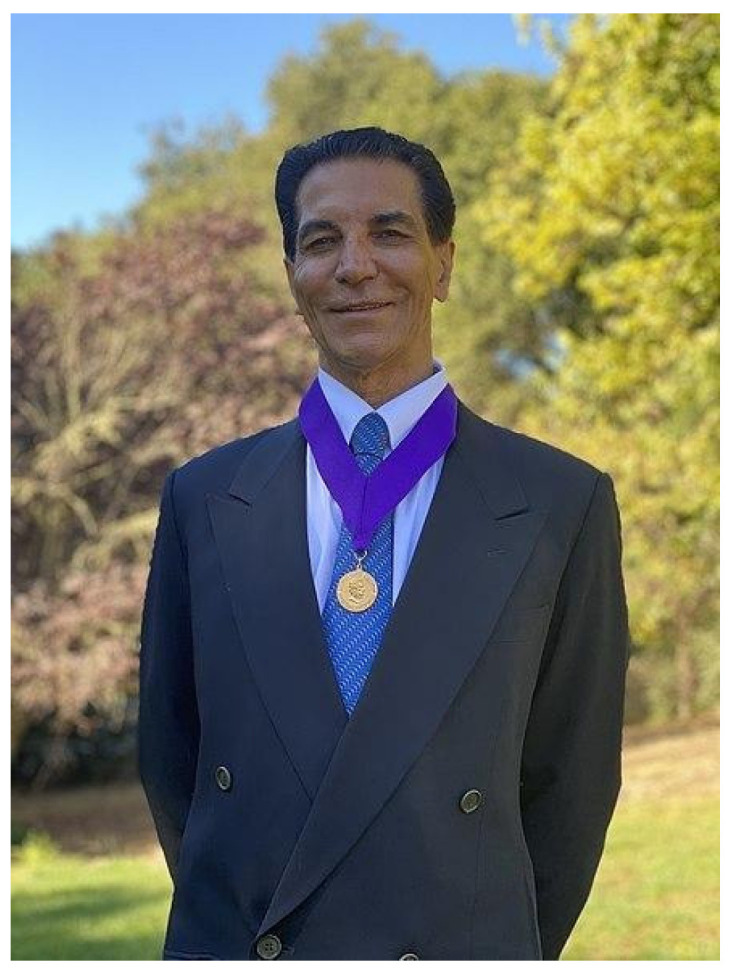
Camran Nezhat, MD with AMA Distinguished Service Award Medal. Images from the personal archive of Dr. Camran Nezhat.

Formal recognition of these contributions culminated in 2020, when Dr. Camran Nezhat received the American Medical Association’s Distinguished Service Award for meritorious service to the art and science of medicine (Figure 6). In announcing the award, AMA President Susan R. Bailey, MD, emphasized that pioneering work in video-assisted surgery had fundamentally reshaped contemporary surgical practice and expanded the ability of surgeons worldwide to deliver safer and more effective care. The citation also acknowledged ongoing leadership in advancing technologies designed to enhance outcomes and reduce patient harm.

Beyond professional recognition, the broader significance of MIS lies in its population-level impact. Over more than four decades, the global shift from open surgical approaches to minimally invasive techniques has represented one of the most significant transformations in the history of surgery [91,92]. Large population-based studies have demonstrated consistent reductions in postoperative mortality, complications, and recovery time compared with open approaches across multiple specialties [91,93]. Many patients have been spared the severe morbidity and prolonged recovery historically associated with open surgery. These outcomes will be discussed in detail in this article.

From a public-health perspective, these outcomes underscore the role of MIS as a large-scale harm-reduction strategy. By decreasing physiological stress, limiting tissue injury, and accelerating recovery, video-assisted approaches have not only improved individual patient outcomes but also reduced cumulative health-system burden. Shorter hospital stays, faster return to productivity, and lower complication rates have translated into measurable benefits for patients, providers, and societies alike [91,92,93].

The magnitude of this impact places MIS among the most consequential medical advances of the modern era. While its development required decades of technical refinement, clinical validation, and institutional adaptation, its ultimate success reflects a simple guiding principle: achieving therapeutic goals while minimizing unintended injury. This principle continues to inform ongoing innovation and serves as a foundation for future advances in surgical care.

### 2.9. Early Complications and Controversies of Minimally Invasive Surgery and the Learning Curve

The rapid and largely unregulated adoption of laparoscopic techniques in the early 1990s was accompanied by a significant and well-documented rise in procedure-specific complications—most strikingly exemplified by bile duct injury (BDI) during laparoscopic cholecystectomy. Prior to the laparoscopic era, BDI during open cholecystectomy occurred at a rate of less than 0.2%, a figure that had been refined over more than a century of surgical experience [94]. Following the introduction of laparoscopic cholecystectomy, this rate climbed to 0.5–1.5% across institutional series in the early 1990s—representing a three- to fivefold increase—and in one statewide audit, the number of bile duct repairs nearly tripled between 1988 and 1992 [95,96]. The primary mechanism underlying this surge was misidentification of the common bile duct for the cystic duct, facilitated by the loss of tactile feedback, the two-dimensional operative view, and the fundamentally different visual perspective and error of perception imposed by the laparoscopic approach—in which the cystic duct is visualised from below rather than from above as in open surgery [97]. Crucially, most surgeons in the early laparoscopic era learned the procedure through one- to three-day postgraduate courses conducted without structured supervision, then returned to independent practice carrying an unresolved learning curve directly into patient care [95]. A national survey of United States surgeons demonstrated that those who trained in laparoscopic cholecystectomy after residency—through these short postgraduate courses—were 39% more likely to sustain at least one biliary injury compared to those trained during a formal supervised residency programme, and 58% more likely to incur two or more injuries over their career [95]. Furthermore, accepting a liberal definition of the learning curve as 200 cases, at least one-third of injuries appeared not to be attributable to inexperience at all, but to systematic technical errors embedded in how the procedure was being practised across the surgical community at large [95]. Sixty-four percent of all major bile duct injuries required biliary reconstruction, carrying substantial long-term morbidity including biliary stricture, cholangitis, secondary biliary cirrhosis, and significantly impaired quality of life [97].

The bile duct injury experience of the laparoscopic cholecystectomy era stands as the most instructive and cautionary case study in the history of surgical innovation, illustrating a recurring tension between the transformative potential of new technology and the risks inherent in its premature or inadequately supervised dissemination. However, it also demonstrates the capacity of the surgical community to course-correct. As structured residency training, simulation-based curricula, and the concept of the critical view of safety became standardised, BDI rates progressively declined. By the mid-2000s, a large population-based study of 156,958 laparoscopic cholecystectomies in New York State documented a BDI rate of 0.08%—now statistically indistinguishable from the historical rate for open cholecystectomy [98]. This trajectory of initial complication elevation followed by iterative improvement has been observed across minimally invasive surgery broadly: in laparoscopic colorectal surgery, anastomotic leak rates and conversion rates declined significantly as structured fellowship training and volume thresholds were established; in minimally invasive cardiac surgery, learning curve analyses suggest that stable morbidity and mortality are achievable after 40–78 supervised cases depending on the procedure [99]; and in minimally invasive spine surgery, systematic reviews demonstrate that most learning curve–associated complications resolve within the first 20–30 consecutive cases performed under appropriate guidance [100]. These data collectively reinforce a principle that has become axiomatic in modern surgical education: the learning curve of a new minimally invasive technique is not merely an institutional inconvenience—it is a measurable, patient-affecting clinical phenomenon that demands structured oversight, credentialling standards, proctored introduction, and honest outcome reporting as prerequisites for responsible adoption [5].

These early complications and concerns led to skepticism from some members of the medical establishment. For example, a letter published in The Lancet questioned whether the growing enthusiasm for laparoscopic surgery represented a “laparoscopic bubble” that might soon burst [101]. Similarly, an editorial in Obstetrics & Gynecology (the Green Journal), the official journal of the American College of Obstetricians and Gynecologists, referred to operative laparoscopy as a possible “technical gimmick” in 1992 [69]. In response to this skepticism, proponents of minimally invasive surgery defended the technique in subsequent correspondence in The Lancet [101]. As clinical evidence accumulated and surgical expertise improved, perceptions gradually changed. Approximately eighteen years later, the same journal revisited the topic in an editorial acknowledging the significant progress and established value of operative laparoscopy [89].

### 2.10. Future Directions, Ethical Imperatives, and the Democratization of Surgical Care

Ongoing collaboration remains central to the continued evolution of MIS. Dr. Camran Nezhat and colleagues continue to work with surgeons and innovators worldwide to expand the scope of video-assisted and robotic approaches, ensuring that advances in technique and technology translate into meaningful improvements in patient care. These collaborations reflect a shared commitment to refining surgical practice through evidence, innovation, and global knowledge exchange.

At present, video-assisted and robotic- assisted MIS represent established standards of care across most surgical specialties. Procedures once considered technically infeasible or unsafe without open access are now routinely performed through minimally invasive approaches. The transition from direct visualization through large incisions to monitor-based operative guidance has fundamentally altered how surgeons conceptualize and execute procedures, enabling safer interventions with reduced physiological burden [57,58,102].

Looking ahead, the integration of artificial intelligence into surgical practice is poised to further enhance the benefits of MIS. AI-driven tools are increasingly being developed to support image interpretation, intraoperative decision-making, and procedural planning. These technologies are expected to augment—not replace—surgeon expertise by providing real-time insights and enhancing precision [103]. Projections suggest that by mid-century, many surgical procedures may be performed by robotic systems operating under human supervision, continuing the trajectory toward safer, more consistent care [104].

The historical arc from Bozzini’s early optical experiments to contemporary intelligent surgical platforms illustrates a continuous progression driven by curiosity, perseverance, and a commitment to reducing patient harm. At each stage, innovation emerged not from technological novelty alone, but from a willingness to challenge entrenched assumptions and reimagine how surgery could be performed more humanely and effectively.

Throughout this journey, a guiding ethical principle has remained constant: the primary obligation of surgery is to heal while minimizing unintended injury. The development and dissemination of video-assisted laparoscopy demonstrated that extensive pathology could be treated through less invasive means without compromising therapeutic effectiveness. By performing complex procedures entirely through video guidance, early adopters opened a pathway for surgeons worldwide to improve outcomes while reducing patient suffering.

This principle is often summarized in a simple inversion of traditional surgical dogma: rather than equating larger incisions with greater expertise, MIS has shown that technical mastery enables smaller incisions and better outcomes. This reframing underscores the importance of skill, preparation, and teamwork over physical exposure alone.

In recent years, efforts have expanded beyond operative technique to include broader initiatives aimed at democratizing access to high-quality care. In 2019, a free global risk-advisory application for endometriosis screening was introduced, providing individuals worldwide with tools to support earlier recognition and referral. Such initiatives reflect an ongoing commitment to applying technological innovation not only within the operating room but also across the continuum of patient care.

Continued collaboration with academic leaders and scientists—including Dr. David K. Stevenson and other respected colleagues—reinforces the belief that progress in medicine must ultimately serve humanity above institutional or disciplinary boundaries [105,106]. By aligning innovation with ethical responsibility, MIS continues to evolve as both a clinical and societal intervention.

## 3. What Minimally Invasive Surgery Has Achieved So Far

In this section, we review the achievements of minimally invasive surgery (MIS) across multiple surgical fields. A search strategy was developed to identify studies comparing outcomes between open surgery and minimally invasive approaches, focusing on mortality, morbidity, and cost-effectiveness.

The search covered a fifty-year period from 1975 to 2025 to ensure broad capture of relevant evidence, ranging from the early development of minimally invasive surgical techniques to the most recent advances in robotic-assisted surgery. Both interventional and observational studies were considered using a mixed-methods approach.

The review includes gynecologic procedures such as hysterectomy, myomectomy, tubal surgery, adnexal surgery, endometriosis surgery, sacrocolpopexy, and gynecologic cancer surgery. In general surgery, the procedures reviewed include cholecystectomy, appendectomy, colorectal surgery for colon cancer, foregut surgery (fundoplication), bariatric surgery, inguinal and ventral hernia repair, and emergency general surgery. Urologic procedures include radical prostatectomy, partial nephrectomy, radical nephrectomy, radical cystectomy, and endourologic procedures such as ureteroscopy (URS) and percutaneous nephrolithotomy (PCNL). Thoracic surgery procedures include lobectomy for non–small cell lung cancer (NSCLC), sublobar resection, diagnostic thoracoscopy, pleural disease management, thymectomy, mediastinal mass resection, esophagectomy, and decortication for empyema.

### 3.1. Volume of Surgeries: MIS vs. Open Surgery

**Figure 7:**
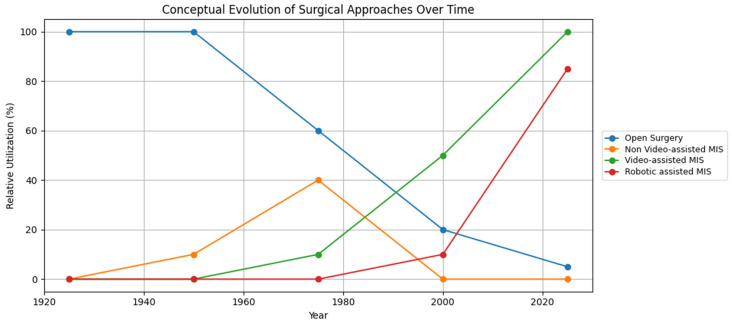
Conceptual illustration of the global transition from open surgery to minimally invasive surgery (MIS) between 1900 and 2025. The figure demonstrates the progressive dominance of MIS, particularly after the widespread adoption of video assisted MIS and advanced surgical technologies, with open surgery now largely reserved for select emergency and complex cases [4,18,73,78,110,111,112,113,114].

Over the last century—and most dramatically since the late 1980s—surgical practice has undergone a global shift from open surgery to MIS. As illustrated in Figure 7, by approximately 2020–2025, MIS accounts for an estimated 80–90% of elective procedures across many surgical domains, while open surgery has declined to approximately 10–20% of cases, largely reserved for trauma, advanced malignancy, or complex emergency situations. This transition has been driven primarily by the widespread adoption of video assisted MIS, followed by the introduction of robotic platforms and advanced energy devices, which together enabled surgeons to perform increasingly complex operations through minimally invasive approaches [1,2,8,107].

Procedures once considered impractical or unsafe without large incisions—such as colectomy, hysterectomy, nephrectomy, and prostatectomy—are now routinely performed laparoscopically or robotically with outcomes comparable or superior to open surgery [1,2,8,107]. These developments have fundamentally altered surgical paradigms, establishing MIS as the default approach rather than the exception across most specialties. As shown in Figure 7, MIS has become the predominant surgical approach worldwide [108,109].

### 3.2. Surgeon Preference and Practice Patterns

Surgeon preference has shifted decisively toward MIS over the past several decades, reflecting both technological advancement and accumulated clinical experience. Key drivers of this shift include superior visualization through video assisted MIS and magnification, improved ergonomics—particularly with robotic platforms—lower complication rates, and increasing patient demand reinforced by institutional standards and quality metrics. In gynecology, general surgery, and urology, MIS is now the preferred approach for most benign conditions and an expanding number of oncologic procedures, as reflected in contemporary training curricula, board certification requirements, and hospital credentialing practices [4,115,116].

**Figure 8:**
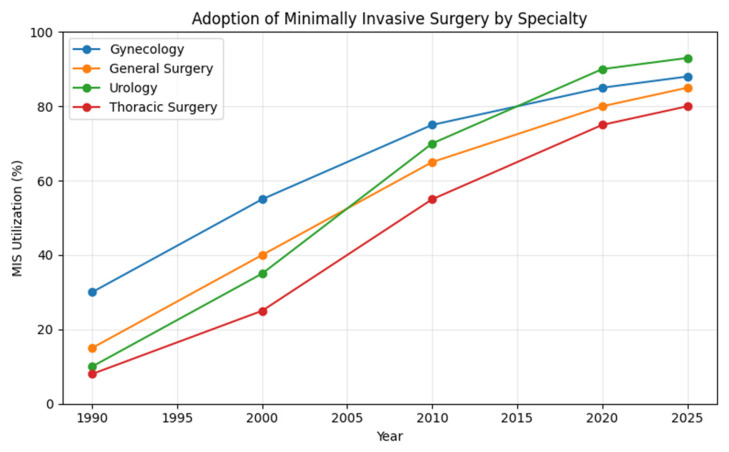
Trends in the adoption of minimally invasive surgery (MIS) across major surgical specialties from 1990 to 2025. Gynecology (blue) established early leadership through the pioneering work of Semm and the widespread conversion of gynecologic oncology procedures. General Surgery (orange) expanded rapidly in the 1990s following the standardization of laparoscopic cholecystectomy. Urology (green) shows marked acceleration in the early 2000s, driven by the disruptive introduction and diffusion of robotic platforms that transformed prostate cancer surgery. Thoracic Surgery (red) demonstrates a later but steady rise, reflecting the progressive maturation of VATS and subsequent robotic- assisted thoracic approaches as techniques and technology became more standardized [117,119,120,121,122,123,124,125,126,127,128,129,130,131,132,133,134,135].

The adoption of robotic surgery further accelerated this transition, especially in urology and gynecologic oncology, by reducing technical barriers associated with conventional MIS, such as limited degrees of freedom and the complexity of intracorporeal suturing [117]. As illustrated in Figure 8, gynecology emerged as an early pioneer in MIS adoption, with other specialties following as technology matured and surgeon expertise expanded [6]. Importantly, MIS is now largely surgeon-driven rather than technology-driven, with surgeons preferentially selecting minimally invasive approaches whenever clinically feasible [92,118].

### 3.3. Mortality Reduction

**Figure 9:**
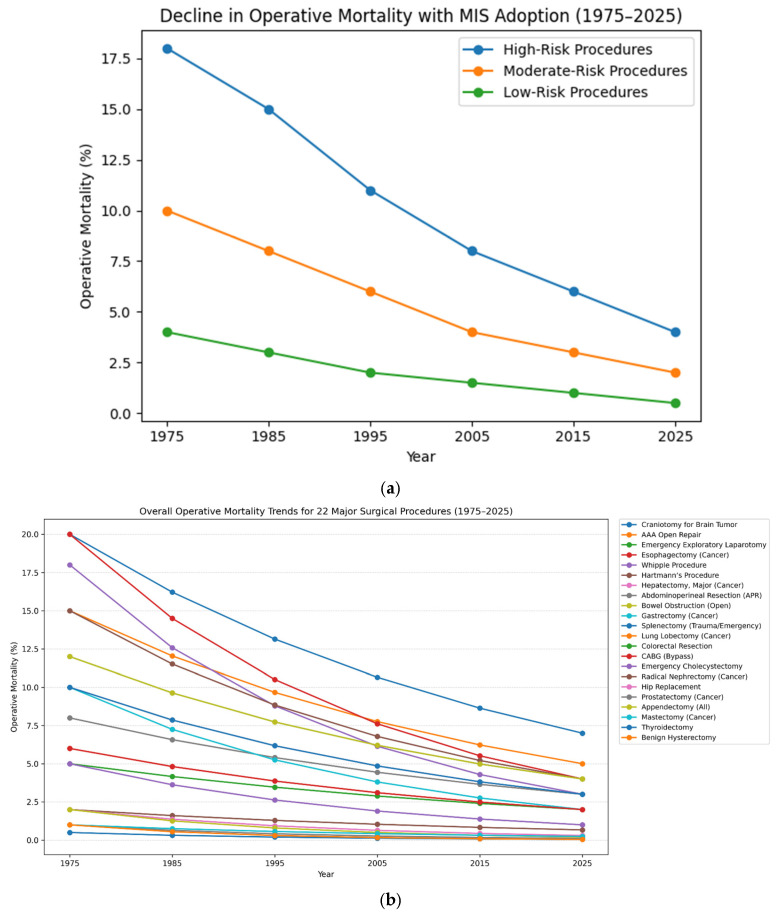
(**a**) Conceptual trends showing declining operative mortality rates for high-risk, moderate-risk, and low-risk surgical procedures between 1975 and 2025. The reduction in mortality parallels the integration of minimally invasive techniques, endovascular therapies, and improved perioperative care pathways. High-Risk procedures (e.g., pancreatectomy, esophagectomy, aortic aneurysm repair) show the steepest decline, supported by longitudinal volume-outcome data. Moderate and Low-Risk procedures (e.g., hysterectomy, colectomy, appendectomy) demonstrate a steady convergence toward minimal mortality, driven by the adoption of laparoscopic and robotic standards. The influence of surgical volume and center specialization is a critical cofactor in these improved survival rates [1,2,116,117,136,137,138,139,140,141,142,143,144]. (**b**) Overall operative mortality trends for 22 major surgical procedures, 1975–2025. The figure demonstrates a consistent decline in perioperative mortality across high-risk, moderate-risk, and lower-risk surgical procedures over five decades. This improvement coincides with advances in perioperative care and, notably, the progressive adoption of minimally invasive and video-assisted surgical techniques, which reduced physiologic stress, surgical trauma, and postoperative complications. Values represent evidence-informed historical trends derived from published surgical series and population-based studies [1,2,116,117,136,137,138,139,140,141,142,143,144].

The mortality trend data spanning 1975 to 2025 demonstrate a consistent and procedure-wide decline in perioperative mortality, with particularly pronounced improvements following the widespread introduction of MIS (Figure 9). High-risk “giant” procedures—including pancreaticoduodenectomy, esophagectomy, open abdominal aortic aneurysm repair, and craniotomy—have experienced approximately two- to fourfold reductions in operative mortality over this period. These improvements are largely attributable to reduced physiologic stress, decreased blood loss, and enhanced perioperative recovery facilitated by shorter operative times and earlier mobilization [136].

For lower-risk and commonly performed procedures such as appendectomy, hysterectomy, and cholecystectomy, operative mortality in elective settings has declined to near-zero levels in contemporary practice [136,137,138,139]. Importantly, these gains reflect not only technical refinement but also the biologic advantages inherent to MIS, including attenuated inflammatory responses and fewer cardiopulmonary complications. Collectively, MIS has contributed to one of the most significant reductions in surgical mortality observed in modern medicine, as demonstrated by the substantial decline in operative mortality over time shown in Figure 9a.

Figure 9b illustrates the long-term decline in operative mortality across 22 major surgical procedures between 1975 and 2025, reflecting one of the most significant safety advancements in modern surgery. While improvements in anesthesia, critical care, and perioperative management have contributed to this trend, the timing and magnitude of mortality reduction closely parallel the widespread adoption of minimally invasive and video-assisted surgical techniques [1,2,140]. The most substantial absolute mortality reductions are observed in historically high-risk procedures, including esophagectomy, pancreaticoduodenectomy (Whipple procedure), craniotomy, and open abdominal aortic aneurysm repair, where reduced tissue trauma, lower blood loss, and faster postoperative recovery have translated into meaningful survival benefits [136,141,142,143]. Importantly, even lower-risk, high-volume operations—such as appendectomy, hysterectomy, prostatectomy, and thyroidectomy—now demonstrate near-zero operative mortality in elective settings, underscoring the cumulative population-level impact of MIS [116,117,139,144]. Collectively, these trends suggest that MIS has altered not only surgical access but also the fundamental biology of postoperative recovery, leading to durable improvements in surgical outcomes across specialties.

### 3.4. Morbidity and Complication Reduction

**Figure 10:**
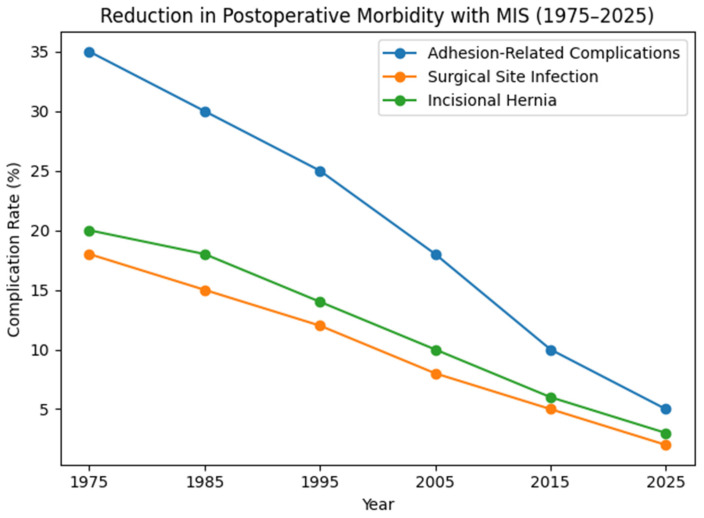
Long-term reduction in major postoperative complications—including adhesion-related morbidity, surgical site infection, and incisional hernia—following the transition from open surgery to minimally invasive approaches. Smaller incisions and reduced tissue trauma have fundamentally altered postoperative recovery [145,146,147,148,149].

Beyond mortality, MIS has led to marked reductions in postoperative morbidity. The Surgical Complications Reduction Atlas (1975–2025) illustrates sharp declines in adhesion-related bowel obstruction, incisional hernia formation, surgical site infection, pulmonary complications, venous thromboembolism, and procedure-dependent complications such as anastomotic leaks (Figure 10). Adhesion-related morbidity following laparotomy has decreased substantially with MIS adoption, while incisional hernias have declined due to smaller fascial defects. In addition, postoperative ileus and pulmonary complications have become less frequent as a result of reduced pain, earlier ambulation, and faster return of bowel function [145,146,147,148].

Across multiple specialties and procedures, minimally invasive approaches are associated with ~40–60% lower overall morbidity/complication rates compared with open surgery in comparative analyses and meta-analyses [149]. These findings highlight that smaller incisions have fundamentally altered postoperative biology and recovery, extending far beyond cosmetic benefits. The marked decline in postoperative morbidity following the widespread adoption of MIS is clearly illustrated in Figure 10.

### 3.5. Cost Effectiveness

Although minimally invasive and robotic approaches may involve higher upfront equipment and operative costs, extensive health economic analyses have demonstrated that MIS is cost-effective—and often cost-saving—when the full episode of care is considered. Reduced hospital length of stay, lower complication rates, fewer readmissions, and faster return to work and productivity collectively offset initial expenditures. Large database studies have reported net cost savings of approximately 10–30% per case for procedures such as colorectal surgery, hysterectomy, bariatric surgery, and urologic operations [150,151,152,153].

**Figure 11:**
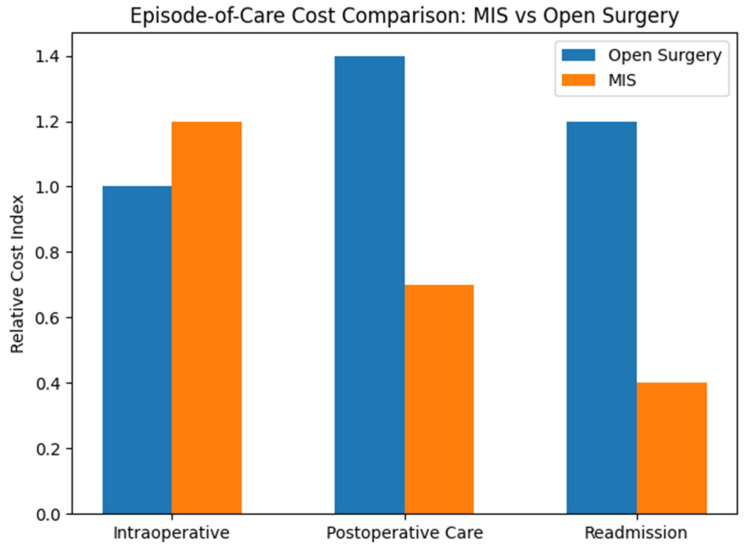
Comparison of relative episode-of-care costs for open surgery and minimally invasive surgery (MIS). The data illustrates a fundamental redistribution of resources: while MIS is associated with higher intraoperative expenditures (driven by instrumentation and operating room time), these costs are offset by substantial reductions in postoperative care and readmission expenses. Systematic reviews suggest that net cost savings are most profound when MIS significantly reduces length of hospital stay and complication-related readmissions [150,151,152,153].

As shown in Figure 11, MIS shifts healthcare costs away from prolonged postoperative care and complication management toward operative precision, resulting in improved value at the system level. Despite higher upfront operative costs, MIS demonstrates superior cost efficiency over the complete episode of care.

### 3.6. Procedure-Specific Impact of Minimally Invasive Surgery

#### 3.6.1. Gynecology

##### Overview

**Figure 12:**
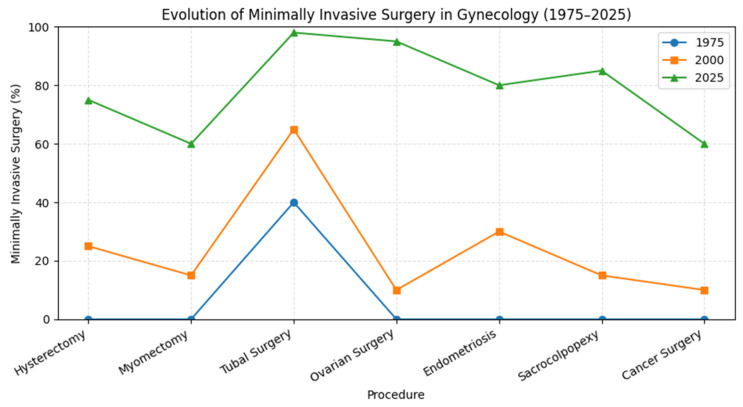
Evolution of Minimally Invasive Surgery (MIS) in Gynecology (1975–2025). Stacked bar chart illustrating the progressive shift from open surgery (red) to MIS (green) across major gynecologic procedures, including hysterectomy, myomectomy, tubal surgery, ovarian surgery, endometriosis, sacrocolpopexy, and cancer surgery. The figure compares three time points (1975, 2000, and 2025), highlighting the substantial increase in MIS adoption over time and the corresponding decline in open surgical approaches [154,155,156,157,158,159,160,161,162,163,164,165,166].

Over the past five decades, MIS has profoundly reshaped gynecologic practice. Figure 12 illustrates the progressive evolution and adoption of minimally invasive techniques across gynecologic procedures and shows the proportion of open versus minimally invasive surgical approaches for selected gynecologic procedures at three time points (1975, 2000, and 2025). These data reflect the progressive replacement of open surgery with MIS over time, with variability by procedure type. The magnitude of benefit varies by procedure complexity, oncologic context, and patient risk profile [154,155,156,157,158,159,160,161,162,163,164,165,166].

##### Benign Hysterectomy

Benign hysterectomy is among the most extensively studied applications of MIS and demonstrates the clearest population-level shift away from open surgery. Large U.S. cohort analyses show a substantial decline in abdominal hysterectomy from 53.6% to 40.1% between 2007 and 2010, alongside an increase in laparoscopic hysterectomy from 24.3% to 30.5% and a rapid rise in robotic-assisted hysterectomy from 0.5% to 9.5% [153,167].

These trends align with surgeon preference data. International surveys indicate that when surgeons select a hysterectomy route for themselves or family members, the majority prefer total laparoscopic hysterectomy (59%) or vaginal hysterectomy (19%), whereas only 5% favor an abdominal approach [168].

Perioperative mortality for benign hysterectomy is exceedingly low and does not significantly differ by surgical route, a finding consistently reported in randomized trials and meta-analyses, though these studies are underpowered to detect mortality differences as a primary outcome [169]. In contrast, morbidity outcomes clearly favor MIS. A landmark meta-analysis comparing total laparoscopic hysterectomy with abdominal hysterectomy demonstrated a weighted mean reduction in estimated blood loss of approximately 183 mL, with a modest increase in operative time of about 22 min and no statistically significant difference in major complication rates [169].

From a health economic perspective, although MIS—particularly robotic-assisted hysterectomy—incurs higher operating room costs, these are offset by shorter hospital stays, fewer wound complications, and reduced downstream resource utilization, rendering MIS cost-effective across the full episode of care [153,169].

##### Myomectomy

The adoption of MIS for myomectomy has been more heterogeneous and remains strongly influenced by fibroid size, number, location, fertility considerations, and surgeon expertise. Unlike hysterectomy, no single national dataset comprehensively captures longitudinal MIS adoption trends for myomectomy.

Nevertheless, contemporary meta-analyses consistently demonstrate perioperative advantages of laparoscopic myomectomy over abdominal myomectomy, including reduced blood loss, lower rates of postoperative ileus, and a shorter length of stay by approximately 1.6 days, despite an increase in operative time of roughly 16 min [170]. Mortality after myomectomy is exceptionally rare and is not reported as a differentiating outcome. Importantly, laparoscopic myomectomy is associated with higher direct procedural costs, reflecting longer operative times and specialized equipment use [170]. Thus, MIS offers clear recovery benefits, while cost-effectiveness remains procedure- and system-dependent.

##### Tubal Surgery for Ectopic Pregnancy and Sterilization

In tubal surgery, particularly for ectopic pregnancy, MIS became the preferred approach for hemodynamically stable patients as early as the late 1980s and 1990s. Surgical approach remains primarily dictated by patient stability, with laparotomy reserved for ruptured ectopic pregnancy or significant hemorrhage. Mortality is uncommon and more closely related to rupture timing and hemorrhage severity than surgical route.

A notable procedure-specific morbidity associated with salpingostomy is persistent ectopic pregnancy. Early comparative cohorts reported higher rates following laparoscopic salpingostomy compared with laparotomy (approximately 15.5% vs. 1.8%), likely reflecting technical limitations during the initial adoption of minimally invasive approaches [171]. Cost analyses generally favor MIS due to shorter hospitalization and faster recovery, although absolute cost estimates vary across healthcare systems.

##### Benign Ovarian and Adnexal Surgery

Benign ovarian and adnexal surgery is now one of the most MIS-dominant areas in gynecology. Nationwide U.S. data from 2010 to 2018 show MIS utilization rates of 89.6% for ovarian cystectomy and 87.3% for oophorectomy, reflecting near-universal adoption [172]. Mortality outcomes are rarely reported due to the extremely low baseline risk.

Large laparoscopic series report high procedural success rates (93.9%), low conversion rates (6.1%), minimal estimated blood loss (mean 89 mL), short hospital stays (mean 0.94 days), and very low perioperative complication rates [173]. Cost advantages largely stem from reduced length of stay and lower conversion rates, favoring MIS in most settings.

##### Advanced Endometriosis and Deep Infiltrating Disease

In high-volume referral centers, advanced endometriosis and deep infiltrating endometriosis (DIE) are managed almost exclusively using MIS approaches, including conventional MIS and robotic-assisted MIS [174,175,176]. Unlike hysterectomy or adnexal surgery, standardized national adoption data comparing MIS with open surgery are limited, as most available evidence derives from specialized, high-volume center–based cohorts [174,175].

Surgeon preference in this context typically reflects a choice between conventional MIS and robotic-assisted MIS rather than open surgery [176]. Mortality is rare, and outcomes primarily focus on perioperative morbidity, symptom control, and long-term reoperation rates [175,176,177]. Comparative studies suggest that robotic-assisted MIS may be associated with reduced postoperative pain and shorter length of hospital stay compared with conventional MIS; however, these potential benefits are offset by increased costs and are highly sensitive to institutional case volume, surgeon experience, and capital investment models [177,178].

##### Urogynecology

Sacrocolpopexy has undergone a gradual transition toward minimally invasive approaches, particularly laparoscopic and robotic techniques, with open surgery now reserved for selected cases [179]. Mortality remains rare, and comparative effectiveness studies primarily focus on perioperative morbidity, recovery, and economic outcomes rather than survival [179,180].

The ACCESS trial provides one of the most robust economic evaluations in this field, demonstrating that robotic-assisted sacrocolpopexy is more costly than laparoscopic sacrocolpopexy in institutions where robotic infrastructure is already established, with an incremental difference of $1155 per case [181]. Clinical outcomes—including blood loss, length of stay, and complication rates—generally favor minimally invasive approaches over open surgery, with comparable long-term anatomic and functional outcomes between laparoscopic and robotic techniques [179,180,181].

##### Gynecologic Oncology

Endometrial cancer staging represents one of the strongest oncologic validations of MIS. Analysis of ACS-NSQIP data demonstrated an increase in MIS utilization from 16% in 2006 to 48% in 2010 [182]. Following the introduction of robotic platforms, a Danish nationwide cohort showed that robotic- assisted MIS accounted for 50.0% of cases, with conventional MIS contributing an additional 22.2% [183].

Importantly, MIS is associated with significantly lower rates of severe postoperative complications, including 30-day mortality. Compared with robotic-assisted MIS, open surgery carried an adjusted odds ratio of 3.87 for severe complications, and 2.58 compared with laparoscopic MIS [183]. MIS also reduced length of stay from 3.8 days to 1.6 days and markedly decreased overall complications (396 vs. 91 events) [182]. From a system-level perspective, each 10% increase in MIS utilization was associated with an estimated savings of $2.8 million and 41 fewer complications [182].

For gynecologic malignancies other than endometrial cancer—including cervical, ovarian, and vulvar cancers—the role of MIS is more heterogeneous and remains highly disease- and stage-dependent. While MIS offers well-established perioperative benefits such as reduced blood loss, shorter hospitalization, and lower wound complication rates, oncologic outcomes must be interpreted with caution [184,185].

In cervical cancer, randomized evidence has demonstrated inferior survival outcomes with minimally invasive radical hysterectomy compared with open surgery in early-stage disease, leading to a paradigm shift favoring laparotomy for oncologic safety [186,187]. In ovarian cancer, MIS is primarily utilized for diagnostic staging and interval debulking in carefully selected cases, whereas open surgery remains the standard approach for primary cytoreduction [188,189,190]. For vulvar cancer, MIS is largely limited to nodal assessment techniques, such as sentinel lymph node biopsy, and select reconstructive applications rather than primary tumor resection [191,192].

Thus, unlike endometrial cancer, MIS in non-endometrial gynecologic cancers is applied selectively, with oncologic safety—rather than perioperative efficiency—serving as the primary determinant of surgical approach [184,190].

Impact of MIS on Mortality and Morbidity in Gynecology Procedures is summarized in Table 2.

#### 3.6.2. General Surgery

**Figure 13:**
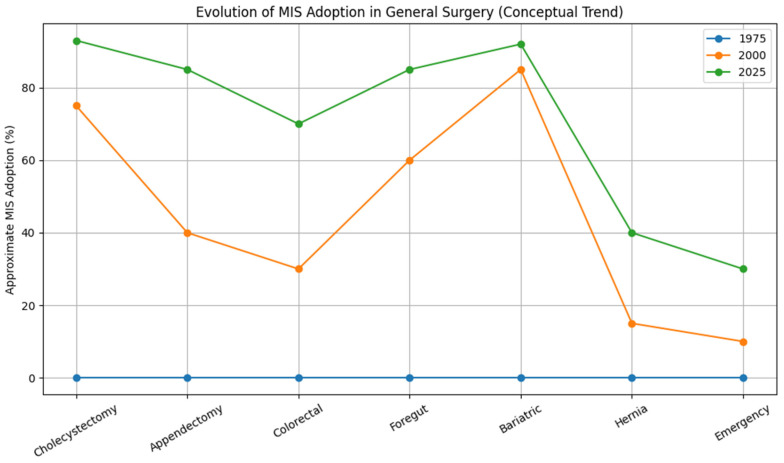
Evolution of minimally invasive surgery (MIS) in general surgery from 1975 to 2025. This figure illustrates the changing proportion of open versus minimally invasive surgical approaches across selected general surgical procedures (including cholecystectomy, appendectomy, colorectal surgery, foregut surgery, bariatric surgery, hernia repair, and emergency surgery) at three time points: 1975, 2000, and 2025. The data demonstrate a progressive shift from open surgery to MIS over time, with varying rates of adoption depending on procedure type [90,203,204,205,206,207,208,209,210,211,212,213,214,215,216].

Over the past five decades, MIS has profoundly reshaped general surgical practice. Figure 13 illustrates the progressive evolution and adoption of minimally invasive techniques across major general surgical procedures and shows the proportion of open versus minimally invasive surgical approaches at three time points (1975, 2000, and 2025). These data reflect the steady replacement of open surgery with MIS over time, with notable variability according to procedure type. As in gynecology, the magnitude of benefit varies by procedure complexity, oncologic context, and patient risk profile [90,203,204,205,206,207,208,209,210,211,212,213,214,215,216].

##### Cholecystectomy

Laparoscopic cholecystectomy represents the earliest and most rapid adoption of MIS in general surgery. Following its introduction in the late 1980s, laparoscopic cholecystectomy increased from <10% of cases in 1990 to >80–90% by the early 2000s, with open cholecystectomy declining to <10% in most developed healthcare systems [107,217]. Surgeon preference overwhelmingly favors laparoscopy due to improved visualization and reduced postoperative pain.

Perioperative mortality for elective cholecystectomy is low (<0.5%) for both approaches; however, MIS is associated with significantly lower surgical site infection rates (2–4% vs. 6–10%), reduced pulmonary complications, and shorter length of stay (median 1–2 days vs. 4–6 days) compared with open surgery [218,219]. Health-economic analyses demonstrate that laparoscopic cholecystectomy is cost-effective despite slightly higher operative costs, due to shorter hospitalization and faster return to work [219].

##### Appendectomy

Laparoscopic appendectomy has progressively replaced open appendectomy, particularly in adults and obese patients. Large meta-analyses show that laparoscopy now accounts for 60–75% of appendectomies in many regions [144]. Surgeon preference reflects lower wound complication rates and improved diagnostic accuracy.

Compared with open appendectomy, laparoscopic appendectomy is associated with lower surgical site infection rates and a shorter length of hospital stay, with a mean reduction of approximately one day [220,221]. Mortality is rare for both approaches and is similar between laparoscopic and open techniques (<0.2%) in large comparative series [221]. Although operative time is modestly longer for laparoscopic appendectomy (approximately 10–15 min), overall episode-of-care costs are reduced, largely due to fewer postoperative complications and shorter hospitalization [221].

##### Colorectal Surgery

MIS adoption in colorectal surgery was initially cautious due to oncologic concerns. However, randomized trials and long-term follow-up studies have firmly established oncologic equivalence. Large trials demonstrate that laparoscopic colectomy now accounts for 45–60% of elective colon cancer resections in high-volume centers [222,223,224].

Compared with open colectomy, MIS is associated with reduced postoperative ileus (approximately 10–15% vs. 20–25%), fewer pulmonary complications (5–8% vs. 12–18%), lower overall morbidity (odds ratio approximately 0.65–0.75), and a shorter hospital stay (median 5–7 vs. 8–10 days), as demonstrated in large meta-analyses and population-level database studies [222,223,225,226]. Several population-based studies demonstrate lower perioperative mortality in elderly patients undergoing laparoscopic colectomy (2.2% vs. 4.5%) [227]. Cost analyses show laparoscopic colectomy to be cost-neutral or cost-saving when reduced complications and shorter LOS are considered [228].

##### Foregut Surgery and Bariatric Procedures

MIS has become the dominant approach for foregut surgery, including antireflux and bariatric procedures. Laparoscopic fundoplication accounts for >90% of antireflux surgeries, with reduced postoperative pain and LOS (mean 2–3 vs. 6–7 days) [229].

Bariatric surgery is now performed almost exclusively using MIS techniques, with >95% of procedures laparoscopic or robotic- assisted in contemporary registries [230]. Despite higher operative costs, MIS bariatric surgery is associated with lower wound complication rates (<2% vs. 8–15% historical open cohorts) and lower perioperative mortality (0.1–0.3% vs. 0.5–1.0%) [230,231].

##### Hernia Surgery

MIS adoption in hernia surgery is procedure specific. Laparoscopic approaches are preferred for bilateral and recurrent inguinal hernias, accounting for 40–60% of repairs in many systems [232]. Compared with open repair, MIS is associated with reduced chronic pain (10–12% vs. 20–30%), faster return to work (mean −7 days), and lower wound infection rates (1–2% vs. 4–6%) [232,233]. Mortality is negligible for both approaches.

##### Emergency General Surgery

The role of MIS in emergency general surgery has expanded substantially. Laparoscopy is now routinely used for appendicitis, perforated ulcer repair, and selected bowel obstructions. In emergency appendectomy, MIS is associated with lower complication rates (12–15% vs. 20–25%) and shorter LOS (−1.3 days) [221,234]. Mortality benefits are indirect and primarily mediated through reductions in infection, pulmonary complications, and physiologic stress.

Across general surgery, MIS procedures incur higher operative costs, particularly with robotic platforms; however, system-level analyses consistently demonstrate cost neutrality or savings when total episode-of-care costs are considered. Reductions in LOS (1–4 days), complications, readmissions, and faster functional recovery offset higher intraoperative expenditures [228]. These findings parallel those observed in gynecology and support MIS as a high-value surgical strategy rather than a cost driver.

Impact of MIS on Mortality and Morbidity in General Surgical Procedures is summarized in Table 3.

#### 3.6.3. Urology

**Figure 14:**
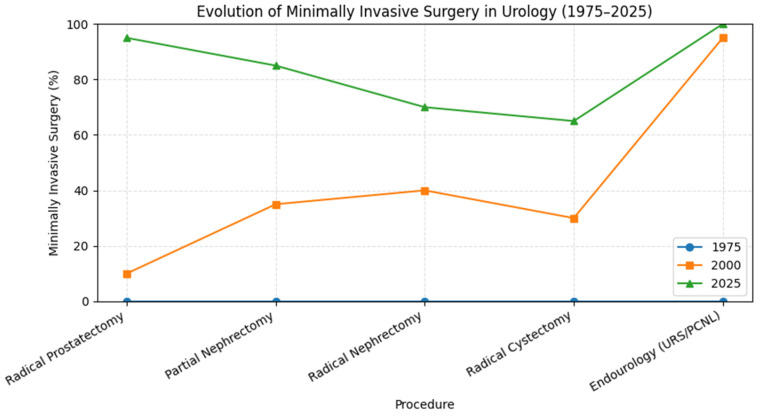
Evolution of minimally invasive surgery (MIS) in urology. Proportion of open versus MIS approaches for selected urologic procedures at three time points (1975, 2000, and 2025). The figure demonstrates a progressive shift from open surgery toward MIS across major urologic procedures, with earlier and more complete adoption in endourologic interventions and variable uptake among reconstructive and oncologic surgeries [45,112,248,249,250,251,252,253,254,255,256,257,258,259,260,261,262,263,264,265,266,267,268,269].

Over the past five decades, MIS has profoundly reshaped urologic practice. Figure 14 illustrates the progressive evolution and adoption of minimally invasive techniques across urologic procedures and shows the proportion of open versus minimally invasive surgical approaches for selected urologic procedures at three time points (1975, 2000, and 2025). These data reflect the progressive replacement of open surgery with MIS over time, with notable variability by procedure type. As in gynecology and general surgery, the magnitude of benefit varies by procedure complexity, oncologic context, and patient risk profile [45,112,248,249,250,251,252,253,254,255,256,257,258,259,260,261,262,263,264,265,266,267,268,269].

##### Radical Prostatectomy

Radical prostatectomy represents the most dramatic example of MIS adoption in urology. Population-based SEER–Medicare analyses demonstrate that minimally invasive radical prostatectomy increased from 9% in 2003 to 42% by 2006, largely replacing open retropubic prostatectomy within a few years of robotic introduction [248]. Subsequent national data show that robotic-assisted radical prostatectomy (RARP) now accounts for >85–90% of prostatectomies in the United States and many other developed healthcare systems [249,250]. This rapid transition reflects surgeon preference for enhanced visualization in the deep pelvis and improved ergonomics.

Perioperative mortality after prostatectomy is low (<0.2%) regardless of approach; however, MIS confers consistent morbidity advantages. Meta-analyses demonstrate significantly lower estimated blood loss with RARP (often 300–600 mL less than open surgery), reduced transfusion rates (1–2% vs. 7–10%), and shorter length of stay (1–2 vs. 3–4 days) [251,252,253]. Operative time is typically longer by 30–60 min, but overall complication rates are comparable or lower with RARP [251,252,253]. Cost analyses show higher operative costs for robotic-assisted surgery, but downstream savings from reduced transfusion, complications, and LOS partially offset these differences [250,254].

##### Partial Nephrectomy (Nephron-Sparing Surgery)

The adoption of MIS for partial nephrectomy has accelerated over the past two decades, particularly with robotic assistance. Contemporary network meta-analyses indicate that robot-assisted partial nephrectomy (RAPN) accounts for approximately 45–50% of partial nephrectomies, compared with 30–35% open and 15–25% laparoscopic approaches in modern series [255]. Surgeon preference increasingly favors RAPN due to improved suturing and ischemia control.

Compared with open partial nephrectomy, RAPN is associated with reduced estimated blood loss (−150 to −300 mL), lower overall complication rates (odds ratio ~0.7), and shorter length of stay (−1.5 to −2 days), while maintaining equivalent oncologic outcomes and renal function preservation [255,256]. Mortality is rare (<0.5%) across all approaches. Costs remain higher for RAPN due to robotic utilization, though reduced LOS and complication rates mitigate total episode-of-care costs in high-volume centers [255,256].

##### Radical Nephrectomy

Laparoscopic radical nephrectomy has largely replaced open nephrectomy for localized renal tumors. Large observational cohorts report MIS utilization rates of 70–80% for radical nephrectomy in contemporary practice [257]. Compared with open surgery, laparoscopic radical nephrectomy is associated with lower postoperative pain, reduced wound complications, shorter LOS (2–3 vs. 5–7 days), and similar cancer-specific survival [257,258]. Mortality is low (<1%) for both approaches, with morbidity benefits favoring MIS.

##### Radical Cystectomy

Radical cystectomy remains one of the most morbid operations in urology, making perioperative outcomes particularly relevant. MIS adoption has been slower than for prostate or kidney surgery; however, robotic-assisted radical cystectomy (RARC) now accounts for 30–40% of cases in high-volume centers [259]. The RAZOR randomized trial demonstrated oncologic non-inferiority of RARC compared with open radical cystectomy [260].

Meta-analyses show that RARC is associated with significantly reduced blood loss and transfusion requirements (risk ratio ~0.58), modestly shorter LOS (~0.5–1 day), and similar overall complication rates, at the expense of longer operative time (+60–90 min) [259,260,261]. Thirty-day mortality remains substantial for cystectomy overall (2–4%) but does not differ significantly by surgical approach in randomized data [260]. Cost analyses indicate higher operative costs for RARC, partially offset by reduced transfusion and LOS [259,260,261].

##### Endourology

Endourologic procedures (Ureteroscopy and Percutaneous Nephrolithotomy) represent an intrinsic form of MIS and have almost completely replaced open stone surgery. Open stone surgery now accounts for <1–2% of cases in most contemporary series [262]. Ureteroscopy and percutaneous nephrolithotomy (PCNL) offer high stone-free rates with low mortality (<0.1%) and acceptable morbidity profiles. Advances such as mini-PCNL have further reduced blood loss, transfusion rates (<1–3%), and LOS compared with standard PCNL [262,263].

Impact of MIS on Mortality and Morbidity in Major Urologic Procedures is summarized in Table 4.

## 4. Thoracic Surgery

### 4.1. Overview

**Figure 15:**
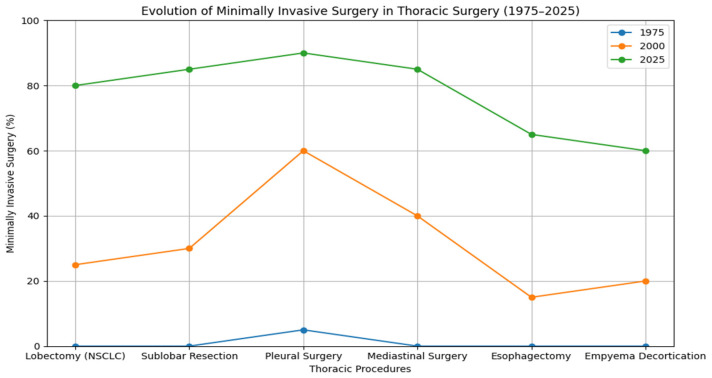
Evolution of minimally invasive surgery (MIS) in thoracic surgery from 1975 to 2025. The schematic illustrates the progressive replacement of open thoracotomy by VATS and robotic approaches for representative thoracic procedures. Adoption is most pronounced for early-stage lung cancer resection, pleural disease, and mediastinal surgery, with more selective uptake for esophagectomy and complex empyema, reflecting differences in technical complexity, oncologic considerations, and patient selection [281,282,283,284,285,286,287,288,289,290,291,292,293].

Over the past three decades, MIS—most prominently video-assisted thoracoscopic surgery (VATS) and, more recently, robot-assisted thoracic surgery (RATS)—has transformed the management of pulmonary, pleural, mediastinal, and esophageal disease. This shift reflects the same principle seen across surgical disciplines: when oncologic and functional outcomes can be maintained, reducing access trauma yields meaningful improvements in perioperative morbidity, recovery, and patient-reported outcomes. The magnitude of benefit varies by resection extent (wedge/segmentectomy vs. lobectomy vs. pneumonectomy), pathology severity (benign vs. malignant, early vs. locally advanced), and technical complexity (adhesions, hilar inflammation, prior radiation/operations) (Figure 15) [281,282,283,284,285,286].

### 4.2. Lung Resection for Suspected or Confirmed Malignancy (NSCLC)

Lung resection—particularly lobectomy and anatomic segmentectomy—represents the most visible and evidence-supported domain of VATS adoption in thoracic surgery. For early-stage non–small cell lung cancer (NSCLC), multiple comparative studies and randomized evidence demonstrate that VATS lobectomy can achieve oncologic adequacy (including lymph node evaluation) while reducing perioperative morbidity, postoperative pain, and length of stay compared with open thoracotomy, without compromising survival in appropriately selected patients [281,282,283].

Procedure-specific benefits are most consistent for outcomes driven by access trauma: reduced pulmonary complications, earlier ambulation, and faster return to baseline function. However, these advantages are sensitive to the learning curve and case selection, as conversion to thoracotomy remains an important safety endpoint in cases with bulky hilar disease, dense fissures, or vascular adhesions [282,284].

RATS has expanded minimally invasive capability in technically demanding hilar and mediastinal dissection, with potential ergonomic and dexterity advantages; however, whether this translates into meaningful clinical superiority over VATS remains debated, and cost considerations are substantial and institution-dependent [283,284,285,289,294].

### 4.3. Sub-Lobar Resection (Segmentectomy/Wedge) and Diagnostic Thoracoscopy

The growth of lung cancer screening and improved imaging has increased detection of small peripheral lesions and ground-glass opacities, accelerating use of VATS diagnostic wedge resection and anatomic segmentectomy for selected early tumors and medically fragile patients [295,296].

In these settings, minimally invasive approaches are often associated with short hospitalization, low pain burden, and rapid functional recovery, while maintaining acceptable oncologic outcomes when anatomic principles and margin goals are respected [297,298]. The primary trade-offs are technical: precise segmental anatomy, intersegmental plane management, and reliable lymph node sampling, all of which require specialized expertise and careful intraoperative decision-making [297,298].

### 4.4. Pleural Disease (Effusions, Biopsy, Pleurodesis, and Pneumothorax)

VATS is widely regarded as the standard minimally invasive platform for pleural disease requiring operative intervention. For recurrent pleural effusion, VATS enables diagnostic biopsy, pleurodesis, and catheter-based strategies with high diagnostic yield and minimal access morbidity [287,288].

For primary spontaneous pneumothorax, VATS bleb/bulla management combined with pleurodesis/pleurectomy has become a dominant approach due to reduced postoperative pain and favorable recovery compared with thoracotomy, though recurrence rates may vary depending on technique, patient factors, and pleural intervention method [287,289]. Overall mortality is extremely low, with outcomes more strongly influenced by underlying lung disease and physiologic reserve than surgical access [287].

### 4.5. Mediastinal Surgery (Thymectomy and Other Mediastinal Masses)

Minimally invasive thymectomy (VATS or robotic) has expanded substantially, particularly for myasthenia gravis and selected thymic tumors, driven by reduced postoperative pain, shorter hospitalization, and improved cosmesis [290]. For carefully selected early-stage thymic epithelial tumors, minimally invasive thymectomy can be performed with acceptable oncologic outcomes in experienced centers, while more invasive tumors with great vessel involvement often remain best served by open approaches [290].

Robotic platforms are commonly adopted in this space due to improved instrument articulation and visualization in the anterior mediastinum, but their incremental value must be balanced against resource utilization and institutional volume [290].

### 4.6. Esophagectomy (Minimally Invasive and Hybrid Approaches)

Although sometimes categorized separately as “upper GI,” esophagectomy is frequently managed within thoracic surgical practice, and minimally invasive esophagectomy (MIE) represents a major milestone in endoscopic-assisted thoracic surgery. Randomized trials and large cohort data support that MIE (fully minimally invasive or hybrid) can reduce pulmonary complications and shorten recovery compared with open esophagectomy, while maintaining oncologic outcomes when performed in high-volume settings [292,299].

However, MIE is among the most technically demanding MIS procedures, with outcomes strongly dependent on surgical volume, team experience, and perioperative pathways. Consequently, broad adoption has been slower and more center-dependent than VATS lung resection [292,299].

### 4.7. Complex Pleural Infection and Decortication (Empyema)

For empyema and complex pleural space infection, VATS can be effective—particularly in earlier stages—by facilitating drainage and decortication with reduced access morbidity [287,293]. In advanced organized empyema, dense adhesions and thick pleural peel may increase conversion rates and prolong operative time; here, the decision between VATS and thoracotomy is often dictated by disease chronicity, imaging, and intraoperative findings rather than a strict preference for MIS [287,293]. Mortality and major complications are driven more by sepsis severity, pulmonary reserve, and timing of intervention than by incision type alone [287,293].

### 4.8. Economic and System-Level Considerations

From a health system perspective, thoracic MIS often shifts cost from inpatient bed-days and postoperative complications toward operating room resource use and capital equipment. In many settings, shorter length of stay and fewer pulmonary complications can offset higher procedural costs for VATS and especially RATS, but this balance is highly sensitive to institutional volume, staffing models, and robotic platform utilization [281,284,285,289]. As with other specialties, the most cost-effective strategy is typically one that matches patient and disease complexity to the least invasive approach that preserves safety and oncologic adequacy, supported by standardized perioperative pathways [281,284,287].

Impact of MIS on Mortality and Morbidity in Major Thoracic Procedures is summarized in Table 5.

## 5. Strengths and Limitations

This review carries several notable strengths. To our knowledge, this is the first comprehensive narrative review documenting the full historical evolution of minimally invasive surgery (MIS), spanning nearly five decades of technological development, clinical adoption, and outcome transformation across multiple surgical specialties. By synthesizing fifty years of evidence—from the earliest laparoscopic experiments to the current era of robotic-assisted and image-guided surgery—this manuscript offers a uniquely broad and longitudinal perspective that has not previously been consolidated in a single work. A further distinguishing strength is that this review is narrated by Dr. Camran Nezhat, the globally recognized pioneer of video-assisted endoscopic surgery, whose firsthand contributions to the development and dissemination of laparoscopic techniques lend this manuscript an unparalleled combination of historical authority, clinical insight, and personal witness to the field’s most transformative milestones. This perspective enriches the historical narrative beyond what a purely bibliographic review could provide and offers a valuable reference for posterity.

Nevertheless, several limitations merit acknowledgment. A number of important and closely related topics fall outside the scope of the present manuscript, including procedure-specific morbidity profiles, oncologic controversies surrounding minimally invasive approaches in various cancer types, debates regarding cost-effectiveness—particularly in the context of robotic surgery—learning curve–associated morbidity, and concerns about the potential overuse of minimally invasive techniques in certain clinical settings. Each of these topics represents a substantial area of ongoing scholarly debate and clinical relevance in its own right. We therefore propose them as important subjects for future publications that could explore these issues with the depth and nuance they deserve.

## 6. Conclusions

MIS has fundamentally reshaped modern surgical practice and, in many settings, has displaced open approaches for a growing proportion of elective procedures. Across diverse surgical specialties, MIS is consistently associated with clinically meaningful improvements in perioperative outcomes—often including fewer complications, shorter hospital stays, faster recovery, and, in appropriately selected patients and procedures, reduced mortality—while also offering the potential for cost savings at the health-system level. MIS is no longer an emerging innovation but a central component of contemporary surgical care. Ongoing advances, including artificial intelligence–assisted guidance, image-fusion technologies, and telesurgical platforms, are likely to further expand its reach and refine precision and safety.

In summary, MIS should be considered the preferred approach when technically feasible and clinically appropriate, while recognizing that patient selection, surgeon expertise, institutional resources, and procedural complexity remain critical determinants of outcomes.

## Figures and Tables

**Table 1:** Pioneers of Minimally Invasive Procedures.

Procedure	Pioneer	Year	Ref.
EARLY ENDOSCOPY ORIGINS			
Lichtleiter (early optical illumination device)	Philipp Bozzini	1806	[14]
GYNECOLOGY & OBSTETRICS			
Diagnostic laparoscopy (human)	Hans Christian Jacobaeus	1910	[2]
Pneumoperitoneum needle	János Veress	1938	[15]
Automated insufflator for laparoscopy	Kurt Semm	1966	[16]
Video-assisted operative laparoscopy	Camran Nezhat	1978	[17]
Hysterectomy (laparoscopic)	Harry Reich	1988	[18]
Myomectomy (laparoscopic)	Kurt Semm	1979	[19]
Laparoscopic-assisted myomectomy	Camran Nezhat	1994	[20]
Tubal/ectopic pregnancy surgery	Patrick Bruhat et al.	1980	[21]
Adnexal surgery	Kurt Semm	1977	[19]
Laparoscopic treatment of extensive endometriosis involving multiple organs	Camran Nezhat	1985	[17]
Sacrocolpopexy (laparoscopic)	Camran Nezhat	1992	[22]
Radical hysterectomy with paraaortic and pelvic node dissection	Camran Nezhat	1989	[23]
Laparoscopic debulking for advanced ovarian cancer	Camran Nezhat	1996	[24]
Laparoscopic removal of dermoid cyst	Camran Nezhat	1989	[25]
Operative laparoscopy during pregnancy	Camran Nezhat	1990	[26]
Laparoscopic management of ovarian remnant syndrome	Camran Nezhat	1990	[27]
Laparoscopic repair of vesicovaginal fistula	Camran Nezhat	1994	[28]
Laparoscopic debulking for advanced ovarian cancer	Camran Nezhat	1996	[24]
Laparoscopic repair of cesarean scar defect (isthmocele)	Camran Nezhat	2003	[29]
GENERAL SURGERY			
Cholecystectomy (first laparoscopic)	Erich Mühe	1985	[30]
Cholecystectomy (popularized)	Philippe Mouret	1987	[31]
Appendectomy (laparoscopic)	Kurt Semm (Germany), Camran Nezhat (USA)	1980	[4,32]
Laparoscopic colectomy	Morris Jacobs et al.	1991	[33]
Laparoscopic Nissen fundoplication	Bernard Dallemagne	1991	[34]
Bariatric surgery (laparoscopic RYGB)	Wittgrove & Clark	1994	[35]
Laparoscopic inguinal hernia repair	Ralph Ger	1982	[36]
TAPP hernia repair	Arregui et al.	1992	[37]
Laparoscopic ventral hernia repair	LeBlanc & Booth	1993	[38]
Laparoscopic bowel resection for endometriosis	Camran Nezhat	1991	[39]
UROLOGY			
Radical nephrectomy (laparoscopic)	Ralph Clayman	1991	[40]
Partial nephrectomy (laparoscopic)	Howard Winfield	1993	[41]
Radical prostatectomy (laparoscopic)	William Schuessler	1992	[42]
Radical cystectomy (laparoscopic)	Parra et al.	1992	[43]
Ureteroscopy (modern)	Reed Goodman	1977	[44]
Percutaneous nephrolithotomy (PCNL)	Fernström & Johansson	1976	[45]
Laparoscopic ureter resection	Camran Nezhat	1992	[46]
Laparoscopic bladder resection	Camran Nezhat	1992	[47]
Laparoscopic ureteroneocystostomy with and without Psoas Hitch	Camran Nezhat	1992	[48]
THORACIC SURGERY			
Diagnostic thoracoscopy	Hans Christian Jacobaeus	1910	[2]
VATS lobectomy	Giancarlo Roviaro	1991	[49]
Thoracoscopic esophagectomy	Alfred Cuschieri	1992	[50]
Minimally invasive esophagectomy (total MIE)	Jim Luketich	1996	[51]
VATS thymectomy	Yim et al.	1992–1993	[52]
Laparoscopic diaphragm resection and repair	Camran Nezhat	1992	[53]
Laparoscopic treatment of thoracic/lung endometriosis	Camran Nezhat	1992	[54]
Laparoscopic treatment of hepatic endometriosis	Camran Nezhat	2005	[55]
VASCULAR SURGERY			
Laparoscopic repair of major retroperitoneal vessels	Camran Nezhat	1997	[56]

**Table 2:** Minimally Invasive Surgery vs. Open Surgery in Gynecology: Comparative Complication Outcomes.

Outcome Measure	Open Surgery	Minimally Invasive Surgery (MIS)	Difference/Effect Size (95% CI)	Study Design	Ref.
**HYSTERECTOMY (Benign Disease)—Laparoscopic vs. Open/Abdominal Hysterectomy**					
Return to normal activities	Later recovery	Faster recovery	Return to normal activities (LH vs. AH) MD −13.6 days (95% CI −15.4 to −11.8); 6 RCTs, 520 women; I^2^ = 71%; low-quality evidence. VH vs. AH also faster (MD −9.5 days, 95% CI −12.6 to −6.4). No difference between LH and VH.	Cochrane SR	[193]
Wound infections	More frequent	Less frequent	Wound infections (LH vs. AH) Fewer wound/abdominal wall infections with LH vs. AH.	Cochrane SR	[193]
Febrile morbidity	More frequent	Less frequent	Febrile morbidity (LH vs. AH) Fewer febrile episodes with both LH vs. AH and VH vs. AH.	Cochrane SR	[193]
Operative time	Shorter	Longer	Operative time (LH vs. AH) Significantly longer operative time with LH vs. AH.	Cochrane SR	[193]
**MYOMECTOMY—Laparoscopic vs. Open Myomectomy**					
Postoperative fever	Higher	Lower	Postoperative fever (laparoscopic vs. open myomectomy) OR 0.44 (95% CI 0.26–0.77, I^2^ = 0%). Risk of postoperative fever approximately 50% lower with laparoscopic myomectomy.	Cochrane SR	[194]
Postoperative pain (6 h)	Higher pain	Lower pain	MD −2.40 (95% CI −2.88 to −1.92); significantly lower pain with MIS	Cochrane SR	[194]
Operative time	Shorter	Longer	Operative time (laparoscopic vs. open myomectomy)	Cochrane SR	[194]
Conversion/surgical complications	Comparable	Comparable	OR 1.11 (95% CI 0.44–2.83); no significant difference	Cochrane SR	[194]
Uterine rupture in subsequent pregnancy	Rare	Rare	Uterine rupture in subsequent pregnancy No studies reported this outcome. The review explicitly notes uterine rupture was not reported in any included trial. Authors call for more studies to assess this. Cannot claim “no significant difference”—there is no data.	Cochrane SR	[194]
**TUBAL SURGERY (Ectopic Pregnancy)—Laparoscopic vs. Open Salpingostomy/Salpingectomy**					
Treatment success	Higher	Lower	Lower with laparoscopic OR 0.28 (95% CI 0.09–0.86). Laparoscopic significantly less successful due to higher persistent trophoblast rate (OR 3.5, 95% CI 1.1–11). However, laparoscopic approach significantly less costly (*p* = 0.03).	Cochrane SR	[195]
Subsequent intrauterine pregnancy rate	~60%	~60%	Non-significant difference in subsequent intrauterine pregnancy rate (laparoscopic vs. open salpingostomy) OR 1.2 (95% CI 0.59–2.5). Non-significant tendency toward lower repeat ectopic pregnancy with laparoscopic (OR 0.47, 95% CI 0.15–1.5).	Cochrane SR	[195]
**OVARIAN/ADNEXAL SURGERY (Benign Tumours)—Laparoscopy vs. Laparotomy**					
Overall adverse events	Higher	Lower	OR 0.30 (95% CI 0.20–0.50); significantly lower overall adverse events with laparoscopy.	Cochrane SR	[196]
Postoperative pain (VAS)	Higher	Lower	Postoperative pain (laparoscopy vs. laparotomy) VAS WMD −2.4 (95% CI −2.7 to −2.0); significant.	Cochrane SR	[196]
Pain-free at day 2	Less likely	More likely	Pain-free at day 2 (laparoscopy vs. laparotomy) OR 7.42 (95% CI 4.86–11.33); significantly more likely to be pain-free with laparoscopy.	Cochrane SR	[196]
Hospital stay	Longer	Shorter	Hospital stay (laparoscopy vs. laparotomy) WMD −2.88 days (95% CI −3.1 to −2.7); significant.	Cochrane SR	[196]
**ENDOMETRIOSIS SURGERY (Bowel/Colorectal Resection)—Laparoscopic vs. Open**					
Overall postoperative complications	Higher	Lower	Overall postoperative complications (laparoscopic vs. open colorectal resection for endometriosis) were significantly higher in open group (*p* = 0.04); Clavien grade 3 complications significantly higher in open group (*p* = 0.03).	RCT	[197]
Intraoperative blood loss	Higher	Lower	Intraoperative blood loss significantly lower with laparoscopic (*p* < 0.05).	RCT	[197]
Digestive symptom improvement (dyschiesia, diarrhoea, bowel pain)	Significant improvement	Significant improvement	Digestive symptom improvement: Dyschesia (*p* < 0.0001), diarrhea (*p* < 0.01), bowel pain/cramping (*p* < 0.0001), asthenia (*p* = 0.0001) all improved significantly in BOTH groups. No significant between-group difference in symptom delta values or quality of life.	RCT	[197]
Gynaecologic symptom improvement (dysmenorrhoea, dyspareunia, back pain)	Significant improvement	Significant improvement	Gynecologic and general symptom improvement: Dysmenorrhea (*p* < 0.0001), dyspareunia (*p* < 0.0001) improved significantly in both groups (gynecologic). Back pain (*p* = 0.001) and asthenia (*p* = 0.0001) also improved (general symptoms). No significant between-group difference.	RCT	[197]
Overall postoperative pregnancy rate	Lower	Higher; cumulative rate 60%	Postoperative pregnancy rate Significantly higher in laparoscopic group (*p* = 0.006). Cumulative pregnancy rate 60% in laparoscopic group.	RCT	[197]
Long-term quality of life and symptom scores (51-month follow-up)	Sustained improvement	Sustained improvement	Long-term QoL, symptoms, and fertility (51-month follow-up) QoL improvement (SF-36) sustained at mean 50.7 months; no difference between short- and long-term or between surgical routes. Fertility: 12/28 (42.9%) wishing to conceive became pregnant, mean time 17 months. No difference in total fertility including IVF between routes, but spontaneous pregnancy occurred only after laparoscopy (*p* = 0.016).	Extended analysis of RCT	[198]
Pregnancy rate—independent cohort (bowel endometriosis)	23.1%	57.6%	Higher pregnancy rate after laparoscopic vs. open bowel resection (no *p* value reported for between-group comparison). Only 26.7% of women ≥35 conceived. Previous laparotomies, age ≥35, uterine adenomyosis, and longer infertility duration associated with lower pregnancy rate.	Prospective cohort	[199]
**GYNAECOLOGIC CANCER—Laparoscopic vs. Open Surgery for Endometrial Cancer**					
Moderate-to-severe complications	21%	14%	Moderate-to-severe complications higher in open surgery (*p* < 0.0001). Intraoperative complication rates similar between groups. Operative time significantly longer with laparoscopy (median 204 vs. 130 min, *p* < 0.001). Lymph node non-removal higher with laparoscopy (8% vs. 4%, *p* < 0.0001). Advanced stage detection equivalent (17% vs. 17%).	RCT (LAP2 trial)	[184]
Hospitalisation >2 days	94%	52%	Hospitalisation >2 days significantly higher in Open surgery (*p* < 0.0001).	RCT (LAP2 trial)	[184]
Blood loss	Higher	Lower	Significantly lower blood loss with laparoscopy. MD −106.82 mL (95% CI −141.59 to −72.06).	Cochrane SR	[200]
Major complications	2.1% (TAH)	1.4% (TLH)	Major complications: TLH 14.6% vs. TAH 14.9% (difference −0.3%, 95% CI −9.1 to 8.5, *p* = 0.95); NS. Minor complications also similar (13.0% vs. 11.7%, *p* = 0.76). TLH associated with significantly less blood loss, less pain medication, shorter hospital stay, and faster recovery (all *p* ≤ 0.002), but longer operative time (*p* < 0.0001).	RCT	[201]
30-day mortality	Very low	Very low	No significant difference in perioperative death rate (within 30 days to 6 weeks) between laparoscopy and laparotomy.	Cochrane SR + RCT (LAP2)	[184,200]
5-year overall survival	89.8%	89.8%	5-year OS nearly identical: 89.8% in both arms. HR for recurrence 1.14 (90% lower bound 0.92; 95% upper bound 1.46)—formal non-inferiority not met per protocol-specified threshold (≤40% increase in recurrence risk), though actual recurrence difference was small (11.4% vs. 10.2% at 3 years, difference 1.14%).	RCT (LAP2)	[202]

**Table 3:** Minimally Invasive Surgery vs. Open Surgery: Comparative Outcomes in General Surgery.

Outcome Measure	Open Surgery	Minimally Invasive Surgery (MIS)	Difference/Effect Size (95% CI)	Study Design	Ref.
CHOLECYSTECTOMY—Laparoscopic vs. Open					
Total complications	~17.5%	~17.0%	~17.0% laparoscopic; ~17.5% small-incision Risk difference −0.01 (95% CI −0.07 to 0.05). No significant difference in mortality or complications for any pairwise comparison (laparoscopic vs. small-incision, laparoscopic vs. open, or small-incision vs. open).	Cochrane overview	[235]
Bile duct injury	0.19%	0.39%	Intraoperative injury (all types) 0.67% (pre-LC era) → 1.33% (post-LC era) Adjusted OR 1.79	Population-based study	[236]
Hospital stay	Longer	Shorter	Hospital stay longer vs. shorter Open vs. laparoscopic: Longer vs. shorter Laparoscopic vs. small-incision: MD −0.72 days (95% CI −1.48 to 0.04); Both MIS techniques significantly shorter stay vs. open (lap vs. open: MD −3.0 days, 95% CI −3.9 to −2.3; small-incision vs. open: MD −2.8 days, 95% CI −4.9 to −0.6).	Cochrane overview	[235]
APPENDECTOMY—Laparoscopic vs. Open					
Overall postoperative complications	17.3%	10.5%	Adjusted complication rates (laparoscopic vs. open appendectomy) Overall complications OR = 0.84 (95% CI 0.75–0.94); infections OR = 0.5 (95% CI 0.38–0.66); GI complications OR = 0.8 (95% CI 0.68–0.96). Shorter hospital stay (2.06 vs. 2.88 days) and higher routine discharge rate (OR = 3.22) all favoring laparoscopic.	Nationwide Inpatient Sample	[205]
Wound infection	Higher	Lower	Wound infection Open: Higher|Laparoscopic: Lower Adults: Peto OR 0.42 (95% CI 0.35–0.51); Children: Peto OR 0.25 (95% CI 0.15–0.42); Consistent benefit favoring laparoscopic in both subgroups.	Cochrane SR	[144]
Intraabdominal abscess	Lower	Higher	Intraabdominal abscess Open: Lower|Laparoscopic: Higher Adults: Peto OR 1.65 (95% CI 1.12–2.43); Children: Peto OR 0.54 (95% CI 0.24–1.22); NS.	Cochrane SR	[144]
Postoperative pain—day 1	Higher	Lower	Postoperative pain—day 1 Open: Higher|Laparoscopic: Lower Adults: MD −7.5 mm on 100 mm VAS (95% CI −10.4 to −4.5); Children: MD −8.0 mm (95% CI −16.5 to 0.5); NS.	Cochrane SR	[144]
Hospital stay	3.8 ± 2.1 days	2.1 ± 1.4 days	Hospital stay Open: Longer|Laparoscopic: Shorter Adults: MD −0.96 days (95% CI −1.23 to −0.70); Children: MD −0.81 days (95% CI −1.01 to −0.62)	Cochrane SR	[144]
30-day mortality	0.24%	0.09%	30-day morbidity (laparoscopic vs. open appendectomy) Overall morbidity: OR 0.60 (95% CI 0.54–0.68) favoring laparoscopic. SSI: OR 0.57 (95% CI 0.50–0.65) favoring laparoscopic. Serious morbidity/mortality: OR 0.87 (95% CI 0.74–1.01); NS. Organ space SSI higher with laparoscopic in complicated appendicitis (OR 1.35, 95% CI 1.05–1.73).	Retrospective cohort	[237]
COLORECTAL SURGERY (Colon Cancer)—Laparoscopic vs. Open Colectomy					
Major complications (Dindo grade 3–4)	20.5%	14.8%	Major complications (laparoscopic vs. open colectomy) 28-day morbidity and mortality did not differ between groups. Laparoscopic associated with less blood loss (median 100 vs. 175 mL, *p* < 0.0001), earlier bowel function recovery, fewer analgesics, and shorter hospital stay (all *p* < 0.0001). Operative time 30 min longer with laparoscopic.	RCT (COLOR trial)	[224]
Intraoperative blood loss	Higher	Lower	Open: Higher|Laparoscopic: Lower Intraoperative blood loss significantly less with laparoscopic colorectal resection.	Cochrane SR	[238]
Hospital stay	Longer	Shorter	Open: Longer|Laparoscopic: Shorter Hospital stay significantly shorter with laparoscopic colorectal resection. Laparoscopic also associated with shorter ileus duration, less pain, lower total/local morbidity, and better quality of life up to 30 days. No difference in general morbidity or mortality.	Cochrane SR	[238]
5-year overall survival	68.4%	69.2%	5-year survival (laparoscopic vs. open colectomy) Disease-free survival: Open 68.4% vs. laparoscopic 69.2% (*p* = 0.94); NS. Overall survival: Open 74.6% vs. laparoscopic 76.4% (*p* = 0.93); NS. Recurrence: Open 21.8% vs. laparoscopic 19.4% (*p* = 0.25); NS. Wound recurrence rare in both groups (0.5% vs. 0.9%). Non-inferiority of laparoscopic confirmed.	RCT (COST trial)	[239]
5-year disease-free survival	Equivalent	Equivalent	3-year disease-free survival (laparoscopic vs. open colectomy) DFS: Laparoscopic 74.2% vs. open 76.2% (*p* = 0.70); difference 2.0% (95% CI −3.2 to 7.2); HR 0.92 (95% CI 0.74–1.15). Overall survival: Laparoscopic 81.8% vs. open 84.2% (*p* = 0.45); HR 0.95 (95% CI 0.74–1.22). Formal non-inferiority boundary (7%) narrowly exceeded (CI upper limit 7.2%), though authors considered difference clinically acceptable.	RCT (COLOR trial)	[240]
FOREGUT SURGERY (Laparoscopic Nissen Fundoplication vs. Open Fundoplication)					
Perioperative complications	Higher	Lower	Perioperative complications (laparoscopic vs. open fundoplication) OR 0.35 (95% CI 0.16–0.75, *p* = 0.007) favoring laparoscopic—65% relative reduction. Also shorter hospital stay (WMD −2.68 days, *p* < 0.0001) and faster return to activity (WMD −7.75 days, *p* = 0.02). However, operative time 39 min longer with laparoscopic and reoperation rate significantly higher (OR 1.79, 95% CI 1.00–3.22, *p* = 0.05). Treatment failure comparable between groups.	Meta-analysis	[241]
Hospital stay	Longer	Shorter by ~2–3 days	Open: Longer|Laparoscopic: Shorter No hospital stay data reported in this trial’s published abstract. Trial was stopped early due to significantly higher primary endpoint rate with laparoscopic (RR 8.8, 95% CI 1.2–66.3, *p* = 0.01), driven by dysphagia (7 vs. 0, *p* = 0.016).	RCT	[242]
Symptom control—10-year follow-up	Comparable	Comparable	10-year follow-up (laparoscopic vs. open Nissen fundoplication) GERD symptom relief comparable (92.4% LNF vs. 90.7% CNF; NS). Heartburn, dysphagia, PPI use (26.6% vs. 22.4%), quality of life, and patient satisfaction all similar. Regurgitation relief slightly better with LNF (98.7% vs. 91.0%, *p* = 0.03). However, reoperation rate significantly higher with open (34.8% vs. 15.2%, *p* = 0.006), driven by incisional hernia repairs (9 vs. 2, *p* = 0.015).	RCT	[243]
BARIATRIC SURGERY—Perioperative Outcomes (Laparoscopic-predominant Era Data)					
Overall complication rate—laparoscopic vs. open bariatric	Significantly higher wound complication, infection, and total morbidity	Significantly lower	Complication rate 17% (95% CI 11–23%). Reoperation rate 7% (95% CI 3–12%). 30-day mortality 0.08% (95% CI 0.01–0.24%) in RCTs. Gastric bypass more effective for weight loss but higher complications. Banding had lower mortality/complications but higher reoperation rate. Sleeve comparable to bypass for weight loss. BMI loss at 5 years: 12–17 points.	Systematic review & meta-analysis	[244]
% Excess weight loss at 1 year	~61%	~62%	Overall %EWL 61.2% (95% CI 58.1–64.4%). By procedure: gastric banding 47.5%, gastric bypass 61.6%, gastroplasty 68.2%, BPD/DS 70.1%. 30-day mortality: restrictive 0.1%, bypass 0.5%, BPD/DS 1.1%. Comorbidity resolution: diabetes 76.8%, hypertension 61.7%, OSA 85.7%, hyperlipidemia improved ≥70%.	Systematic review & meta-analysis	[245]
INGUINAL & VENTRAL HERNIA REPAIR—Laparoscopic vs. Open					
Chronic groin pain (>6 months)	Higher	Lower	Chronic groin pain (inguinal hernia) Open: Higher|Laparoscopic: Lower Persisting pain: Peto OR 0.54 (95% CI 0.46–0.64, *p* < 0.0001). Persisting numbness: Peto OR 0.38 (95% CI 0.43–0.49, *p* < 0.0001). Both significantly favor laparoscopic.	Cochrane SR	[232]
Return to normal activities	Longer	Shorter	Return to normal activities (inguinal hernia) Open: Longer|Laparoscopic: Shorter HR 0.56 (95% CI 0.51–0.61, *p* < 0.0001) favoring laparoscopic, equivalent to approximately 7 days earlier return. However, operative time was 14.8 min longer with laparoscopic, and visceral/vascular injuries were more frequent with laparoscopic approach.	Cochrane SR	[232]
Recurrence at 2 years (inguinal hernia)	0.5%	1.9%	2.2 (95% CI 1.5–3.2) favoring open mesh. Significant interaction by hernia type (*p* = 0.012): primary hernia recurrence 4.0% open vs. 10.1% laparoscopic; recurrent hernia repair similar (14.1% vs. 10.0%). Complications also higher with laparoscopic (39.0% vs. 33.4%, OR 1.3). Laparoscopic had less initial pain and 1 day earlier return to activity.	Cochrane SR	[246]
Recurrence at ≥1 year (ventral/incisional hernia)	Similar	Similar	Open: Similar|Laparoscopic: Similar RR 1.22 (95% CI 0.62–2.38, I^2^ = 0%); NS. No significant difference, though follow-up was less than two years in half of trials. Higher intraoperative enterotomy risk with laparoscopic (Peto OR 2.33, 95% CI 0.53–10.35; NS, based on only 7 events).	Cochrane SR	[247]
Wound infection (ventral hernia)	Higher	Lower	Open: Higher|Laparoscopic: Lower RR 0.26 (95% CI 0.15–0.46, I^2^ = 0%); significant. Most clear and consistent finding in the review. Laparoscopic also shortened hospital stay in 6/9 trials but with heterogeneous data. Laparoscopic associated with higher in-hospital costs.	Cochrane SR	[247]

**Table 4:** Minimally Invasive vs. Open Surgery in Urology: Comparative Complication Outcomes.

Outcome Measure	Open Surgery	Minimally Invasive Surgery (MIS)	Difference/Effect Size (95% CI)	Study Design	Ref.
RADICAL PROSTATECTOMY—Robot-Assisted/Laparoscopic vs. Open Retropubic RP					
Blood transfusion	11.1%	2.7%	OR 0.34 (95% CI 0.28–0.40); significantly lower transfusion rate with RARP. Propensity score-matched, multivariable analysis.	Nationwide Inpatient Sample (NIS)	[252]
In-hospital complications (any)	11.3%	7.4%	OR 0.47 (95% CI 0.31–0.71). Postoperative complications: OR 0.86 (95% CI 0.77–0.96). Both significantly favor RARP. Propensity score-matched.	Nationwide Inpatient Sample (NIS)	[252]
Hospital stay	3.0 days	1.9 days	LOS: OR 0.28 (95% CI 0.26–0.30); significantly lower odds of prolonged stay with RARP. Specific mean days not reported in abstract.	Nationwide Inpatient Sample (NIS)	[252]
Blood transfusion	20.8%	2.7%	Blood transfusion (MIRP vs. RRP) 2.7% MIRP vs. 20.8% RRP (*p* < 0.001); significantly lower with MIRP.	SEER-Medicare retrospective cohort	[253]
Overall complications at 6 weeks	42.4%	38.5%	Open: 9%|RARP: 4% (*p* = 0.052); NS but trending. Intraoperative adverse events: 8% open vs. 2% RARP.	RCT	[270]
Blood transfusion and blood loss	Higher	Lower	RR 0.24 (95% CI 0.12–0.46); low-quality evidence. Approximately 68 fewer transfusions per 1000 men. Overall surgical complications: RR 0.41 (95% CI 0.16–1.04); NS. Serious postoperative complications: RR 0.16 (95% CI 0.02–1.32); NS. Hospital stay: MD −1.72 days (95% CI −2.19 to −1.25). Urinary and sexual QoL: no difference.	Cochrane SR	[271]
Intraoperative blood loss	Higher	Lower	LRP and RALP showed significantly lower blood loss, fewer transfusions, and shorter hospital stay vs. ORP. Total perioperative complications higher for ORP and LRP than RALP. Intraoperative complications lowest for RALP. Multiple specific complications (readmission, reoperation, nerve/ureteral/rectal injury, DVT, pneumonia, wound infection)	Systematic review & meta-analysis	[272]
PARTIAL NEPHRECTOMY—Laparoscopic or Robot-Assisted vs. Open PN					
Overall complications	Higher	Lower	On multivariate analysis, laparoscopic PN associated with shorter operative time (*p* < 0.0001), decreased blood loss (*p* < 0.0001), and shorter hospital stay (*p* < 0.0001). Intraoperative complications comparable. However, laparoscopic PN associated with longer ischemia time (*p* < 0.0001), more postoperative complications especially urological (*p* < 0.0001), and more subsequent procedures (*p* < 0.0001).	Cohort study	[254]
Mean blood loss	Higher	Lower	Significantly lower blood loss with laparoscopic PN on multivariate analysis (*p* < 0.0001).	Cohort study	[254]
Hospital stay	5.2 days	2.9 days	Significantly shorter hospital stay with laparoscopic PN on multivariate analysis (*p* < 0.0001).	Cohort study	[254]
Blood transfusion	Similar	Similar	No significant difference in transfusion rates between robotic and open PN, (OR 0.81, 95% CI 0.54–1.23; *p* = 0.32; 7 comparisons from 6 studies, *n* = 3090). Pooled events: 55/587 RPN (9.4%) vs. 166/2503 OPN (6.6%).	Systematic review & meta-analysis	[273]
Estimated blood loss	Higher	Lower	WMD −106.83 mL (95% CI −176.4 to −37.27; *p* = 0.003); significantly lower EBL with robotic PN.	Systematic review & meta-analysis	[273]
Overall complications	Higher	Lower	OR 0.53 (95% CI 0.42–0.67; *p* < 0.00001); significantly lower perioperative complication rate with robotic PN. RPN also had shorter hospital stay (WMD −2.78 days, *p* < 0.00001) but longer operative time (WMD +40.89 min, *p* = 0.002).	Systematic review & meta-analysis	[273]
Blood transfusion	Higher	Lower	RR 0.64 (95% CI 0.41–0.98; *p* = 0.04); significantly lower transfusion rate with RAPN.	Systematic review & meta-analysis	[274]
Warm ischemia time	Shorter	Longer	WMD +3.65 min (95% CI 0.75–6.56; *p* = 0.01); significantly longer WIT with RAPN in primary analysis. However, sensitivity analysis excluding selection bias showed no difference. RAPN also had lower postoperative complications (RR 0.60, *p* = 0.0002), less EBL (WMD −98.82 mL, *p* < 0.00001), and shorter LOS (WMD −2.64 days, *p* < 0.00001).	Systematic review & meta-analysis	[274]
RADICAL NEPHRECTOMY—Laparoscopic vs. Open Radical Nephrectomy					
Mean blood loss	451 mL	172 mL	*p* < 0.001—significantly lower blood loss with laparoscopic RN. Difference ~279 mL.	Retrospective series	[275]
Hospital stay	5.2 days	3.4 days	*p* < 0.001—significantly shorter hospital stay with laparoscopic RN. Difference ~1.8 days	Retrospective series	[275]
Post-op Pain and Recovery	Higher/longer	Lower/shorter	Pain scores significantly lower with laparoscopic (VAS 3.6 vs. 5.4, *p* = 0.02); no difference at 3 months. Return to normal activities faster (42 vs. 62 days, *p* = 0.04). Blood loss, complications, mortality, and hospital stay (median 4 vs. 5 days, *p* = 0.9) all similar. Operative time similar (105 vs. 93 min, *p* = 0.4).	RCT	[276]
RADICAL CYSTECTOMY—Robot-Assisted Radical Cystectomy (RARC) vs. Open RC					
90-day complications	66%	62%	*p* = 0.7—No significant difference. Trial closed early as interim analysis met futility criteria. Both rates high, reflecting complexity of RC regardless of approach. Hospital stay identical (mean 8 days, *p* = 0.5). 3- and 6-month QoL similar. Cost favored open.	RCT (MSKCC)	[277]
High-grade complications (Clavien ≥ 3)	21%	22%	High-grade complications listed as secondary outcome. Overall grade 2–5 complication rates similar (see above). No significant difference (*p* = 0.9).	RCT (MSKCC)	[277]
Intraoperative blood loss	574 mL	258 mL	Significantly lower blood loss with RARC (*p* = 0.027). However, operative time significantly longer with RARC (*p* < 0.001). Pathologic variables including positive surgical margins and lymph node yields similar.	RCT (MSKCC)	[277]
Adverse Events	Similar	Similar	No significant difference. Non-inferiority of RARC confirmed for 2-year progression-free survival (72.3% vs. 71.6%, difference 0.7%, P-non-inferiority = 0.001). Most common adverse events: UTI (35% RARC vs. 26% open) and postoperative ileus (22% vs. 20%).	RCT (RAZOR trial)	[278]
Intraoperative blood loss	575 mL	258 mL	Significantly lower blood loss with RARC (*p* < 0.0001). RARC also had lower narcotic requirements (89 vs. 147 mg morphine equivalents, *p* = 0.004) and faster return of bowel function (3.2 vs. 4.3 days to first bowel movement, *p* = 0.0008). Operative time longer with RARC (252 vs. 211 min, *p* < 0.0001).	RCT	[279]
Hospital stay	Similar	Similar	No significant difference in hospital stay between RARC and open RC.	RCT	[279]
Overall complications	Similar	Similar	No significant difference in overall complication rate. LN yield noninferior: mean 19 (robotic) vs. 18 (open). RARC favorable for EBL, time to flatus/bowel movement, and narcotic use.	RCT	[279]
Major complications (Clavien ≥ 3)	Higher	Lower	Significantly lower major complications with RARC at both 30 days (10% vs. 30%, *p* = 0.007) and 90 days (17% vs. 31%, *p* = 0.03). Overall 30-day complications also lower (41% vs. 59%, *p* = 0.04).	Prospective cohort	[280]

**Table 5:** Minimally Invasive vs. Open Surgery in Thoracic Surgery: Comparative Complication Outcomes.

Outcome Measure	Open Surgery	Minimally Invasive Surgery (MIS)	Difference/Effect Size (95% CI)	Study Design	Ref.
LOBECTOMY (VATS/Thoracoscopic) vs. Open Thoracotomy for NSCLC					
Overall morbidity	34.7%	26.2%	*p* < 0.0001—significantly lower overall morbidity with VATS. Overall pulmonary complications also lower (12.2% vs. 7.6%, *p* = 0.0001). No difference in operative mortality.	Propensity-matched Analysis; STS database	[300]
Atrial fibrillation	11.5%	7.3%	*p* = 0.0004—significantly lower arrhythmia rate with VATS. Also lower reintubation (1.4% vs. 3.1%, *p* = 0.0046) and blood transfusion (2.4% vs. 4.7%, *p* = 0.0028).	Propensity-matched Analysis; STS database	[300]
Hospital stay	6 days	4 days	*p* < 0.0001—significantly shorter hospital stay with VATS. Chest tube duration also shorter (3.0 vs. 4.0 days, *p* < 0.0001).	Propensity-matched Analysis; STS database	[300]
Mortality at Hospital	1.9%	1.0%	*p* = 0.0201—significantly lower mortality at hospital discharge with VATS. VATS also associated with shorter hospital stay (mean 7.8 vs. 9.8 days, *p* = 0.0003).	Propensity-matched analysis; ESTS database	[301]
Major cardiopulmonary complications (Clavien ≥ 3)	19.6%	15.9%	*p* = 0.0094—significantly lower major cardiopulmonary complications with VATS. Specific complications favoring VATS: atelectasis requiring bronchoscopy (2.4% vs. 5.5%, *p* < 0.0001), initial ventilation >48 h (0.7% vs. 1.4%, *p* = 0.0075), wound infection (0.2% vs. 0.6%, *p* = 0.0218). No difference in postoperative atrial fibrillation (*p* = 0.14).	Propensity-matched analysis; ESTS database	[301]
Overall complications	31.7%	29.1%	*p* = 0.0357—significantly lower total complications with VATS, though absolute difference is modest (2.6%). Hospital stay 2 days shorter with VATS (7.8 vs. 9.8 days, *p* = 0.0003).	Propensity-matched analysis; ESTS database	[301]
Long-term outcomes	Higher	Lower	No significant differences in short-term complications (air leak *p* = 0.71, arrhythmia *p* = 0.86, pneumonia *p* = 0.09, mortality *p* = 0.49). However, VATS associated with reduced systemic recurrence (*p* = 0.03) and improved 5-year mortality (*p* = 0.04). No difference in locoregional recurrence.	Systematic review & meta-analysis	[302]
Perioperative outcomes (VATS vs. muscle-sparing thoracotomy)	Higher	lower	No significant differences in operative time, blood loss, chest tube duration, hospital stay, or postthoracotomy pain. Significantly more complications in thoracotomy group (*p* < 0.05), mostly prolonged air leaks.	RCT	[303]
Hospital stay	7 days	4 days	2-day shorter hospital stay with VATS (*p* < 0.001). Fewer complications with VATS (OR 0.73, *p* = 0.06; trending but NS). No difference in 5-year survival (HR 0.72, *p* = 0.12). 1 postoperative death in each group.	Propensity-matched comparative study	[304]
Postoperative pain and quality of life	More pain; lower QoL	Less pain; better QoL	Clinically relevant pain (NRS ≥3) in first 24 h: VATS 38% vs. thoracotomy 63% (*p* = 0.0012). Over 52-week follow-up, moderate-to-severe pain episodes significantly less frequent with VATS (*p* < 0.0001). EQ5D quality of life significantly better with VATS (*p* = 0.014); QLQ-C30 not significantly different (*p* = 0.13). Surgical complications (grade 3–4) similar between groups.	RCT	[305]
Perioperative outcomes	Higher morbidity; longer stay	Lower morbidity; shorter stay	Operative mortality similar (VATS 0% vs. open 1.6%, *p* = 1.0). VATS had less atelectasis requiring bronchoscopy (0% vs. 6.3%, *p* = 0.035), fewer chest tubes >7 days (1.5% vs. 10.8%, *p* = 0.029), shorter hospital stay (5 vs. 7 days, *p* < 0.001), and shorter operative time (117.5 vs. 171.5 min, *p* < 0.001). Lymph node yield and margin status similar.	Secondary propensity-adjusted analysis of RCT	[306]
2-year survival and perioperative outcomes	86%	87%	2-year survival: MIS 87% vs. open 86% (*p* = 0.04). No difference in nodal upstaging or 30-day mortality. Shorter hospital stay with MIS (5 vs. 6 days, *p* < 0.01). However, 30-day readmission higher with MIS (5% vs. 4%, *p* < 0.01). VATS vs. robotic: no significant differences in upstaging, mortality, or survival.	Population-based propensity-matched analysis	[307]
PLEURAL DISEASE (Spontaneous Pneumothorax)—VATS vs. Open Thoracotomy					
Recurrence	Similar	Similar	No significant difference between open and VATS (*p* = 0.15). First episode recurrence: open 7.7% vs. VATS 10.3%. Conservative treatment had much higher recurrence (56.4%).	Retrospective cohort	[308]
Hospital stay	Longer 11.5 days	Shorter 4.1 days	*p* < 0.001—significantly shorter hospital stay with VATS.	Retrospective cohort	[308]
THYMECTOMY—Robot-Assisted/Thoracoscopic (VATS) vs. Open (Transsternal/Thoracotomy)					
Intraoperative blood loss	354.5 mL	100.9 mL	*p* < 0.001—significantly lower blood loss with robotic thymectomy.	Propensity-matched Analysis	[309]
Hospital stay	6.4 days	2.5 days	*p* < 0.001—significantly shorter hospital stay with robotic thymectomy.	Propensity-matched Analysis	[309]
Overall complications	12%	1%	*p* = 0.002—significantly lower postoperative complications with robotic thymectomy. 3-year overall survival identical (100% vs. 100%, *p* = 0.88). Freedom from recurrence similar (92% vs. 99%, *p* = 0.12).	Propensity-matched Analysis	[309]
Perioperative outcomes and oncologic safety—thymic malignancies	More blood loss; longer stay	Less blood loss; shorter stay	Significantly less blood loss and shorter hospital stay with MIS. No significant differences in operative time, respiratory complications, cardiac complications, or overall complications.	Systematic review & meta-analysis	[310]
Overall complications	Similar	Similar	No significant difference in postoperative cardiac, respiratory complications, or vocal cord paralysis (*p* = 0.60). MIS had significantly less blood loss (*p* < 0.01) but no difference in transfusion requirements (*p* = 0.16) or operative time (*p* = 0.88).	Retrospective Cohort	[311]
Hospital stay	5.3 days	2.7 days	Significantly shorter hospital and ICU lengths of stay with MIS (*p* < 0.01 for both). Higher blood loss associated with higher ICU admission rates (*p* < 0.01).	Retrospective Cohort	[311]
Hospital stay	6.2 days (mean)	2.9 days (mean)	*p* = 0.0001—significantly shorter with VATS. Remained significant after propensity score adjustment (*p* = 0.0043).	Retrospective Cohort	[312]
MEDIASTINAL MASS RESECTION—VATS vs. Open Thoracotomy					
Overall morbidity	Higher	Lower	*VATS is associated with reduced surgical trauma and may reduce morbidity compared with open approaches.*	Narrative review	[313]
Hospital stay	Longer	Shorter	*VATS is associated with shorter hospital stay compared with open approaches.*	Narrative review	[313]
ESOPHAGECTOMY—Minimally Invasive Esophagectomy (MIE) vs. Open Esophagectomy					
Pulmonary infections	29% (2-week); 34% (in-hospital)	9% (2-week); 12% (in-hospital)	2-week: RR 0.30 (95% CI 0.12–0.76, *p* = 0.005). In-hospital: RR 0.35 (95% CI 0.16–0.78, *p* = 0.005). Primary endpoint of the trial.	RCT (TIME trial)	[292]
Intraoperative blood loss	475 mL	200 mL	Significantly lower blood loss with MIE (*p* value and specific volumes from full text). Vocal cord paralysis also significantly lower with MIE (2% vs. 14%, *p* = 0.012).	RCT (TIME trial)	[292]
Hospital stay	14 days	11 days	Significantly shorter hospital stay with MIE. Quality of life at 6 weeks also significantly better with MIE. Specific values from full text.	RCT (TIME trial)	[292]
Overall morbidity and mortality	Similar	Similar	Overall morbidity (62.2%) and all-cause mortality (3.8%) equivalent between groups. MIE had higher reoperation (9.9% vs. 4.4%, *p* < 0.001) and empyema (4.1% vs. 1.8%, *p* < 0.001). Open had higher wound infection (6.3% vs. 2.3%, *p* < 0.001), transfusion (18.7% vs. 14.1%, *p* = 0.002), and ileus (4.5% vs. 2.2%, *p* = 0.002).	STS National Database	[314]
Hospital stay and operative time	10.0 days; 312 min	9.0 days; 443 min	Shorter hospital stay with MIE (*p* < 0.001). However, significantly longer operative time (*p* < 0.001).	STS National Database	[314]
Anastomotic leak	15.5%	21.2% (MIE)	*p* = 0.028—significantly HIGHER anastomotic leak with MIE. Reintervention also higher with MIE (28.2% vs. 21.1%, *p* = 0.017).	Propensity-matched cohort	[315]
Pulmonary complications	34.2%	35.6%	Pulmonary complications NS (*p* = 0.669). Overall complications NS (*p* = 0.468). Mortality NS (3.0% vs. 4.7%, *p* = 0.209).	Propensity-matched cohort	[315]
Major pulmonary complications	42.9% MPPC; 59.3% overall morbidity; 7.1% mortality	15.7% MPPC; 35.7% overall morbidity; 1.4% mortality	MPPC: *p* < 0.001. Overall morbidity: *p* < 0.001. In-hospital mortality: *p* = 0.018. All significantly favor HMIO. HMIO independently protective against MPPC and ARDS on multivariable analysis. Lymph node yield and survival equivalent.	Cohort Study	[316]
Overall complications and respiratory outcomes	Higher respiratory complications; higher ICU admission	Lower respiratory; higher GI complications	Overall postoperative complications comparable (*p* = 0.34). Respiratory complications higher in OO (*p* = 0.008). ICU admission higher in OO (*p* = 0.02). However, GI complications higher in MIO (*p* = 0.005), specifically gastroparesis (*p* = 0.004). Hospital mortality comparable (*p* = 0.66). Less blood loss with MIO (*p* = 0.01) but longer operative time (*p* = 0.001). 5-year survival equivalent.	Retrospective Cohort	[317]
5-year overall survival	Lower	Higher	HR 0.82 (95% CI 0.76–0.88); 18% lower 5-year all-cause mortality with MIE. Limited heterogeneity (I^2^ = 12%). No confounding on meta-regression adjusting for age, physical status, tumor stage, and neoadjuvant/adjuvant therapy.	Systematic review & meta-analysis	[318]

## Data Availability

No new data were created or analyzed in this study.

## Abbreviations

The following abbreviations are used in this manuscript:

MIS	Minimally invasive surgery
VATS	Video-assisted thoracoscopic surgery
RATS	Robot-assisted thoracic surgery
NSCLC	Non–small cell lung cancer
VAS	Visual analog scale
ICU	Intensive care unit
SAGES	Society of American Gastrointestinal and Endoscopic Surgeons
FLS	Fundamentals of Laparoscopic Surgery
